# Unraveling the molecular relevance of brain phenotypes: A comparative analysis of null models and test statistics

**DOI:** 10.1016/j.neuroimage.2024.120622

**Published:** 2024-04-20

**Authors:** Zhipeng Cao, Guilai Zhan, Jinmei Qin, Renata B. Cupertino, Jonatan Ottino-Gonzalez, Alistair Murphy, Devarshi Pancholi, Sage Hahn, Dekang Yuan, Peter Callas, Scott Mackey, Hugh Garavan

**Affiliations:** a Shanghai Xuhui Mental Health Center, Shanghai 200232, China; b Department of Psychiatry, University of Vermont College of Medicine, Burlington VT, 05401, USA; c Department of Psychiatry, University of California San Diego, La Jolla, CA, USA; d Division of Endocrinology, The Saban Research Institute, Children’s Hospital Los Angeles, Los Angeles, CA, USA; e Department of Mathematics and Statistics, University of Vermont College of Engineering and Mathematical Sciences, Burlington VT, 05401, USA

**Keywords:** Imaging-transcriptomics, Imaging-derived phenotypes, Gene set analysis, Competitive null models, Self-contained null models

## Abstract

Correlating transcriptional profiles with imaging-derived phenotypes has the potential to reveal possible molecular architectures associated with cognitive functions, brain development and disorders. Competitive null models built by resampling genes and self-contained null models built by spinning brain regions, along with varying test statistics, have been used to determine the significance of transcriptional associations. However, there has been no systematic evaluation of their performance in imaging transcriptomics analyses. Here, we evaluated the performance of eight different test statistics (mean, mean absolute value, mean squared value, max mean, median, Kolmogorov-Smirnov (KS), Weighted KS and the number of significant correlations) in both competitive null models and self-contained null models. Simulated brain maps (*n* = 1,000) and gene sets (*n* = 500) were used to calculate the probability of significance (Psig) for each statistical test. Our results suggested that competitive null models may result in false positive results driven by co-expression within gene sets. Furthermore, we demonstrated that the self-contained null models may fail to account for distribution characteristics (e.g., bimodality) of correlations between all available genes and brain phenotypes, leading to false positives. These two confounding factors interacted differently with test statistics, resulting in varying outcomes. Specifically, the sign-sensitive test statistics (i.e., mean, median, KS, Weighted KS) were influenced by coexpression bias in the competitive null models, while median and sign-insensitive test statistics were sensitive to the bimodality bias in the self-contained null models. Additionally, KS-based statistics produced conservative results in the self-contained null models, which increased the risk of false negatives. Comprehensive supplementary analyses with various configurations, including realistic scenarios, supported the results. These findings suggest utilizing sign-insensitive test statistics such as mean absolute value, max mean in the competitive null models and the mean as the test statistic for the self-contained null models. Additionally, adopting the confounder-matched (e.g., coexpression-matched) null models as an alternative to standard null models can be a viable strategy. Overall, the present study offers insights into the selection of statistical tests for imaging transcriptomics studies, highlighting areas for further investigation and refinement in the evaluation of novel and commonly used tests.

## 1. Introduction

The whole-brain gene expression maps of the Allen Human Brain Atlas (AHBA) have enabled investigation into the spatial association between ex vivo transcriptional and in vivo imaging-derived patterns (Hawrylycz et al., 2012). Linking regional transcriptional profiles and interregional variations in cortical measures has informed our understanding of the putative molecular architectures (e.g., biological processes, cell-type specificity) underlying cortical phenotypes related to health and disease (Arnatkeviciute, et al., 2021; Norbom, et al., 2021; Paus, 2022). For instance, a popular integrative analysis that correlated a set of transcriptional profiles specific to molecular features of interest with a single imaging-derived phenotype showed the potential to reveal possible molecular architectures associated with brain development and disorders (Hess, et al., 2018; Parker, et al., 2020; Patel, et al., 2021, 2022, 2020; Patel, et al., 2019; Pecheva, et al., 2020; Romme, et al., 2017; Shin, et al., 2018; Vidal-Pineiro, et al., 2020). Furthermore, recent studies have extended its utility to understanding specific cognitive functions (Hansen, et al., 2021; Lotter, et al., 2023), further expanding the scope of its applications. This field has been evolving rapidly, presenting us with additional challenges in methodology. While studies have provided reassuring validation (Hansen, et al., 2021; Martins, et al., 2021; Seidlitz, et al., 2020), there still exists a lingering skepticism related to our understanding of the importance of key methodological options.

Permutation tests, in which a test statistic derived from an empirical model is tested against null models have been widely used in gene set analyses that examine the correspondence between transcriptional profiles of prior gene sets and imaging-derived phenotypes. Importantly, the specific approach used to create null models may lead to different findings. For example, when null models are created by resampling genes (i.e., randomly selecting an equally sized set of genes), the null hypothesis is that observed correlations within the gene set are no different than would be observed among these other randomly selected gene sets (aka. competitive null hypothesis) (Goeman and Bühlmann, 2007; Khatri, et al., 2012). Recent studies have advocated for spatially-informed null models (i.e., spinning brain regions) when examining the associations between molecular profiles and imaging-derived phenotypes given that they preserve the spatial dependence of imaging phenotypes (Burt, et al., 2020; Fulcher, et al., 2021; Wei, et al., 2022). Here, the null hypothesis is that observed correlations within the gene set are no different than would be observed with a random distribution of effects across brain regions, albeit while maintaining the spatial autocorrelation of the brain map (aka. self-contained null hypothesis) (Goeman and Bühlmann, 2007; Khatri, et al., 2012).

The terms “self-contained” and “competitive” are used to describe different approaches in a gene set analysis (Goeman and Bühlmann, 2007). In general, self-contained null models examine a set of genes in isolation, without considering the rest of the genes. Thus, these models focus on evaluating the significance of a set of genes on their own, independent of the other genes. In contrast, competitive null models compare a set of genes against all other genes. These models prioritize distinguishing the most important pathway from the rest. Competitive and self-contained null models have their advantages and disadvantages (Khatri, et al., 2012; Maleki, et al., 2020) and both have been used in previous imaging transcriptomic studies (Hess, et al., 2018; Martins, et al., 2021; Parker, et al., 2020; Patel, et al., 2021, 2020, 2019; Pecheva, et al., 2020; Romme, et al., 2017; Shin, et al., 2018; Vidal-Pineiro, et al., 2020). For instance, the competitive null models have been widely used in identifying the transcriptional profiles associated with age- or psychopathology-related cortical differences (Hess, et al., 2018; Parker, et al., 2020; Patel, et al., 2021, 2022, 2020; Patel, et al., 2019; Pecheva, et al., 2020; Romme, et al., 2017; Shin, et al., 2018; Vidal-Pineiro, et al., 2020), while others advocated for or used the self-contained null models (Alexander-Bloch, et al., 2018; Cao, et al., 2023; Fulcher, et al., 2021; Giacomel, et al., 2022; Hansen, et al., 2021; Martins, et al., 2021).

Another important element in the permutation test, the test statistic, is a summary score that represents all correlations within the prior gene set. Various test statistics have been used in the analysis of neuroimaging transcriptomics. For instance, the mean correlation between the brain map and the transcription maps of all genes within the prior gene set (i. e., Mean) is commonly used (Parker, et al., 2020; Patel, et al., 2021, 2022, 2020; Patel, et al., 2019; Shin, et al., 2018; Vidal-Pineiro, et al., 2020). Notably, other test statistics are also available to summarize the correlations within the prior gene set, such as the rank-based enrichment scores (i.e., Kolmogorov-Smirnov; KS) (Giacomel, et al., 2022; Subramanian, et al., 2005), mean squared correlation (Pecheva, et al., 2020), max mean (Efron and Tibshirani, 2007) and so forth (Ackermann and Strimmer, 2009). Some of these test statistics have been used in imaging-transcriptomics studies in combination with either competitive (Pecheva, et al., 2020) or self-contained null models (Cao, et al., 2023; Giacomel, et al., 2022). Although several test statistics have been evaluated in previous studies (Ackermann and Strimmer, 2009; Mathur, et al., 2018), there has been no systematic evaluation of their performance using imaging-derived phenotypes, an evaluation that would assist researchers in making informed choices about statistical tests based on an understanding of their strengths and limitations in the context of the imaging transcriptomics.

The present study utilized the AHBA transcriptomic data that were mapped to the left Desikan-Killiany cortical regions and examined the performance of sixteen statistical tests in detecting the associations between prior gene sets and an imaging-derived brain map. Specifically, we systematically evaluated the performance of eight test statistics in the competitive and self-contained null models (i.e., sixteen statistical tests in total) using simulated gene sets (*n* = 500) and brain maps (*n* = 1000). The probability of identifying significant associations (Psig) between the gene sets and brain maps was calculated to quantify the performance of the statistical tests. Then, we explored potential factors that would affect the performance of statistical tests. Comprehensive supplementary analyses with various configurations were performed to validate the main results.

## 2. Material and methods

### 2.1. Transcriptional profiles

The Allen Human Brain Atlas (http://www.brain-map.org) provided human postmortem brain gene expression maps employing a whole-brain microarray survey on six donors (Hawrylycz, et al., 2012). Microarray data underwent preprocessing using the “abagen” toolbox (Markello, et al., 2021a), following established recommendations (Arnatkevic̆iūtė, et al., 2019; Markello, et al., 2021b). Preprocessing steps involved intensity-based filtering of microarray probes, selecting representative probes for each gene across both hemispheres, assigning microarray samples to brain parcels from the Desikan-Killiany parcellations, normalizing and aggregating the data within parcels and across multiple donors. Specifically, microarray probes were reannotated using data provided by (Arnatkevic̆iūtė, et al., 2019). Probes not matched to a valid Entrez ID were discarded. Probes were filtered based on their expression intensity relative to background noise (Quackenbush, 2002). Probes with intensity less than the background in >=50 % of samples across donors were discarded. When multiple probes indexed the expression of the same gene, the probe with the most consistent pattern of regional variation across donors (differential stability) was selected and used. The MNI coordinates of tissue samples were updated to those generated via non-linear registration using the Advanced Normalization Tools (ANTs, https://github.com/chrisfilo/alleninf). Samples were assigned to brain regions by minimizing the Euclidean distance between the MNI coordinates of each sample and the nearest surface vertex. Samples, where the Euclidean distance to the nearest vertex was more than 2 standard deviations above the mean distance for all samples belonging to that donor, were excluded. Sample-to-region matching was constrained by hemisphere and gross structural divisions (cortex, subcortex/brainstem, and cerebellum), such that, for example, a sample in the left cortex could only be assigned to an atlas parcel in the left cortex. All tissue samples not assigned to a brain region in the provided atlas were discarded. Inter-subject variation was addressed by normalizing tissue sample expression values across genes using a robust sigmoid function (Fulcher, et al., 2013). Normalized expression values were then rescaled to the unit interval. Gene expression values were then normalized across tissue samples using an identical procedure. Samples assigned to the same brain region were averaged separately for each donor and then across donors, yielding a regional expression matrix. Genes with a similarity greater than a threshold of r_donors_ > 0.4 across donors were included, resulting in a total of 6513 genes across cortical regions (i.e., background genes) for the subsequent analysis. The pre-processed data, provided by the ENIGMA Toolbox (Larivière, et al., 2020), can be accessed at (https://github.com/saratheriver/enigma-extra). There was no tissue sample that corresponded to the right frontal pole and the right temporal pole in the Desikan-Killiany atlas. Therefore, only transcriptional profiles of the left hemisphere were included in the analysis. A sensitivity analysis with a lower threshold of r_donors_ > 0.2 was performed to assess the robustness of the findings.

### 2.2. Gene set analysis

**Fig. 1.**
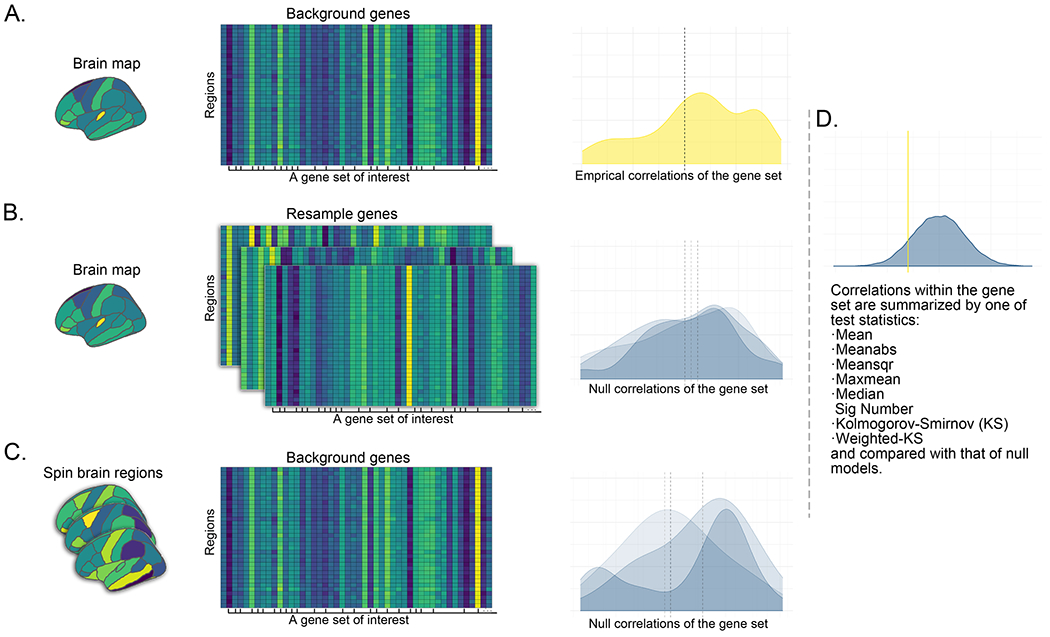
A typical gene set analysis in which researchers examine the association between a derived brain map and the transcriptomic profiles of a set of genes. **A.** The derived brain map is correlated with the transcriptional profiles of background genes (i.e., the 6513 available genes). The density plot illustrates the distribution of the empirical correlations for a gene set of interest. The y-axis represents the estimated probability density of observing a correlation at the x-axis. **B.** Competitive null models built by resampling genes. Three repetitions (i.e., iterations) are illustrated. For each repetition, the original brain map is correlated with background genes with labels shuffled. The density plots represent the resultant null distributions. **C.** Self-contained null models built by spinning brain regions. Three repetitions (i.e., iterations) are illustrated. For each repetition, the original background genes are correlated with the brain maps whose regions are shuffled while preserving their spatial information (i.e., spinning the brain phenotype). The density plots represent the resultant null distributions. **D.** The empirical correlations within a prior gene set are summarized by one of the following test statistics: Mean, Meanabs, Meansqr, Maxmean, Median, Sig Number, KS and Weighted KS, and compared against that from either competitive or self-contained null models. The yellow vertical line represents the summarized value of the empirical correlations. A total of 5000 null models are built. For each repetition, the null correlations are summarized, and the density plot shows the distribution of the summarized values of the null correlations obtained from either the competitive or self-contained null models.

Fig. 1 shows a typical gene set analysis in which we examined the association between a derived brain map and the transcriptomic profiles of a set of genes. As shown in Fig. 1A, the derived brain map was correlated with the transcriptional profiles of background genes (i.e., the 6513 available genes). Then, the empirical correlations within the prior gene set were summarized using a selected test statistic (Fig. 1D) and compared against the same statistic from either competitive or self-contained null models.

Table 1 shows the test statistics that have been examined in the current study. We chose test statistics mainly based on their relevance and common usage in the fields of gene set analysis and imaging transcriptomics, as well as our interests. The mean (Mean), median (Median) and mean squared (Meansqr) correlations have been used in previous studies (Parker, et al., 2020; Patel, et al., 2021, 2020, 2019; Pecheva, et al., 2020; Shin, et al., 2018; Vidal-Pineiro, et al., 2020). The mean absolute (Meanabs) value captured the correlation strength, and the max mean (Maxmean) emphasized the most pronounced average correlation, highlighting dominant patterns in the data (Efron and Tibshirani, 2007). The Kolmogorov-Smirnov (KS) test and its weighted variant were commonly used in gene set analyses to evaluate the enrichment of specific gene sets within ranked lists of genes (Subramanian, et al., 2005). Specifically, the KS test computed the cumulative sum of ranked correlations between all genes and brain profiles. For each gene, this cumulative sum increased if the gene was part of the gene set and decreased otherwise. The adjustment made for each gene, whether an increment or decrement, was weighted by 1. The weighted KS test followed a similar procedure but the adjustment made for each gene was weighted by the correlation strength of each gene. This method has been recently adopted in the field of imaging transcriptomics (Giacomel, et al., 2022). Furthermore, as the cluster size based significance testing was a widely used strategy in neuroimaging studies (Hayasaka and Nichols, 2003), we were interested in assessing the applicability of a similar strategy (i.e., testing the number of significant correlations; Sig Number) within the context of imaging transcriptomics. For this test statistic, multiple comparisons within the gene sets were corrected using false discovery rate (FDR) approach (Benjamini and Hochberg, 1995), and correlations with FDR-corrected *p* < 0.05 within gene sets were deemed as significant.

For the competitive null models (Fig. 1B), the original brain map was correlated with background genes (i.e., 6513 genes) with labels shuffled. For the self-contained null models (Fig. 1C), the original background genes were correlated with the brain maps whose regions were shuffled while preserving their spatial information (i.e., spun brain maps) (Váš et al., 2018). For each type of the null models, the null correlations within the prior gene set were summarized using the selected test statistic and the procedure was repeated 5000 times, resulting in 5000 null distributions of the selected test statistic.

### 2.3. Simulation of brain maps

To measure the spatial autocorrelation in the brain map, Moran’s I and its expected value were calculated (Paradis and Schliep, 2019):

$$I=\frac{N}{W}\frac{\sum_{i}\sum_{j}w_{ij}(x_{i}-\bar{x)}(x_{j}-\bar{x)}}{\sum_{i}(x_{i}-\bar{x{)}^{2}}}$$

$$E(I)=\frac{-1}{N-1}$$

where *N* is the number of the regions (i.e., 34 Desikan regions) indexed by *i* and *j*, *x* is the variable of interest, *W_ij_* is the matrix of spatial weights, and *W* is the sum of all *W_ij_*. The weight matrix was set as the inverse Euclidean distance between the centroids of regions *i* and *j*. *E(I)* is the expected value of Moran’s I under the null hypothesis of no spatial autocorrelation. For the 34 Desikan regions, the expected value of Moran’s I where there was no spatial autocorrelation was −1/34–1=−0.03. A Moran’s I greater or smaller than the expected value indicated a positive or negative spatial autocorrelation.

First, we examined the performance of Moran’s I by simulating 1000 random brain maps by drawing each regional value independently from a uniform distribution ranging from −1 to 1. In this simulation, the averaged Moran’s I (i.e., a measure of spatial autocorrelation) was centered around the expected value of no spatial autocorrelation (i.e., −1/(34–1)=−0.03), suggesting the Moran’s I was a good indicator of the spatial dependence for the Desikan atlas (see Analysis S18). Note that the Global Moran’s I has been used in previous studies as a measure for spatial correspondence (Markello and Misic, 2021). To determine a proper Moran’s I to simulate, we derived the Moran’s I from 12 statistical maps of cortical thickness differences associated with psychiatric and neurological disorders comprising a total of 14,886 cases and 20,962 controls from seven ENIGMA disease-related working groups as well as a cortical map of standard loadings of the first principal component derived from cortical thickness of 24,750 adult participants (see Table S1). The average of Moran’s I across several realistic brain maps was 0.03, therefore, the desired center of the subsample was set as 0.03. Then, 1000 random brain maps with a specific spatial autocorrelation were simulated by randomly drawing a subset of 1000 brain maps with Moran’s I centered at a desired value with a standard deviation of 0.001 from 100,000 random brain maps where each regional value was independently drawn from a uniform distribution ranging from −1 to 1. Simulated brain maps with Moran’s I centered around 0.01 and 0.02 were also examined in the supplementary analysis. As shown in Analysis S18, this subsampling approach was computationally efficient for simulating random Desikan atlas maps with spatial autocorrelation, and it offered better control over the desired Moran’s I in comparison to the Gaussian random fields approach as described in the previous studies (Burt, et al., 2020; Markello and Misic, 2021). As shown in Analysis S21, the main results were replicated when simulating the random brain maps with each regional value independently drawn from a Gaussian distribution centered at 0 with a standard deviation of 1.

### 2.4. Simulation of gene sets

The simulated gene sets that varied in their numbers of genes (20 to 200 with an increment of 20, thus 10 sets in total) were created by randomly selecting genes from the full list of available genes (i.e., 6513 genes). To avoid sampling bias, the process was repeated 50 times, which resulted in 500 different simulated gene sets in total (i.e., 10 simulated gene sets with different sizes × 50 times). Realistic gene sets were also examined in the supplementary analysis.

### 2.5. Analytical strategies

**Fig. 2.**
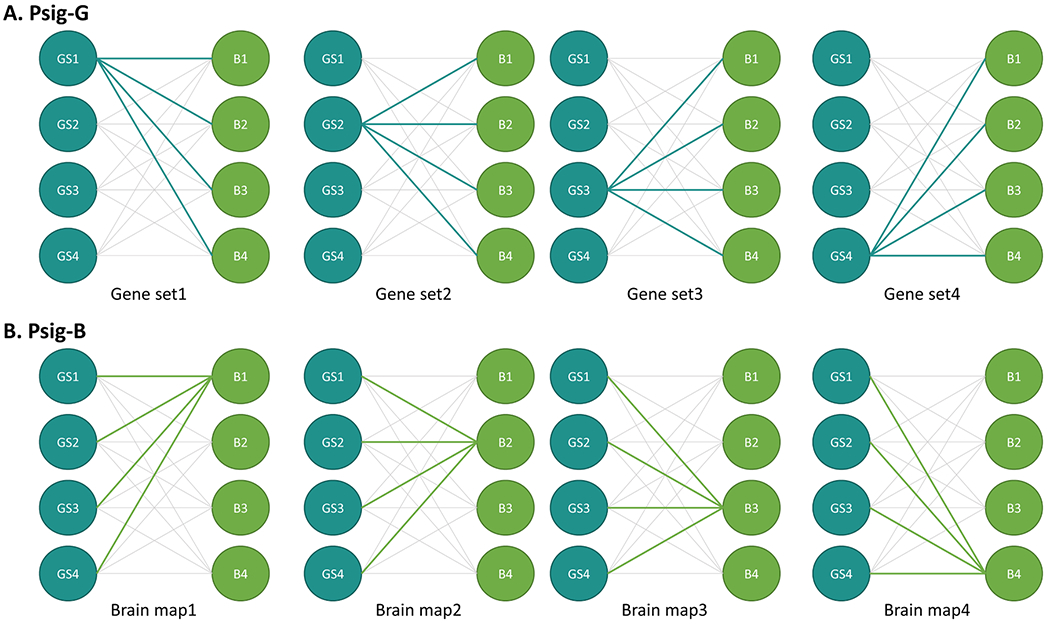
Illustration of calculation of Psig-G (A) and Psig-B (B). Blue circles represent simulated gene sets, green circles represent simulated brain maps, and the lines between them denote one of the statistical tests examined. The panels show examples of the calculation of Psig-G for four gene sets (**A**) and Psig-B for four brain maps (**B**). Psig-G represents the proportion of simulated brain maps that significantly correlate with a gene set, while Psig-B represents the proportion of gene sets that significantly correlate with a brain map.

To quantify the performance of the different statistical tests, the probability of observing significant correlations (Psig) between gene sets and brain maps was calculated for each statistical test. Specifically, this was separately done by calculating Psig for each gene set (Psig-G) and each brain map (Psig-B). Both Psig-B and Psig-G provided information about the likelihood of observing significant correlations between gene sets and brain maps. As demonstrated in Fig. 2, the major difference between Psig-B and Psig-G rested on what was held constant (gene set vs. brain map) when aggregating the significant counts, which provided different perspectives on the results. The Psig-G was calculated as the proportion of the randomly generated brain maps that significantly correlated with a certain gene set. For example, if 680 out of 1000 simulated brain maps were found significantly correlated with a gene set, the Psig-G associated with the statistical test was calculated as 680/1000=0.68 for the gene set. The Psig-B was calculated as the proportion of the gene sets that significantly correlated with a certain brain map. For example, if 100 of 500 gene sets were found significantly correlated with a brain map, the Psig-B associated with the statistical test was calculated as 100/500=0.2 for the brain map. A higher Psig-G indicated that a larger proportion of the randomly generated brain maps showed a significant correlation with the gene set. A higher Psig-B suggested that a larger proportion of the randomly generated gene sets showed a significant correlation with the brain map. When interpreting the results, it is important to keep in mind that the aim was not to find the measure with the lowest Psig, as this could indicate an overly conservative test. Rather, the desirable outcome would be a Psig around 0.05 with a tight standard error.

The concern with the competitive null models based on resampling genes was that the randomly selected genes were unlikely to have expression profiles (e.g., co-expression) that were similar to those of the original gene set (Fulcher, et al., 2021; Khatri, et al., 2012). Here, we examined the relationship between gene co-expression and the Psig-G. Specifically, co-expression was calculated as the averaged pairwise correlations among all gene expression maps of the gene set and this average was correlated with the Psig-G using a linear regression model. The resulting *t*-statistics associated with the co-expression quantified the contribution of the co-expression to the Psig-G and the R-squared of the model quantified the variances in Psig-G explained by the co-expression.

For Psig-B, we found that some randomly simulated brain maps showed a high Psig-B when using the self-contained null models (see Fig. 5). For example, 13 simulated brain maps were significantly correlated with all the simulated gene sets using the Meanabs as the test statistic in the self-contained null models. By inspecting the histograms of the correlations between each of these brain maps and the background genes (i.e., the 6513 available genes), we noted numerous bimodal distributions. Then we hypothesized that the bimodality of the correlation distribution was not accounted for in the self-contained null models and thus contributed to inflated Psig-B. Therefore, we explored the relationship between the bimodality of the background correlation and Psig-B. Several measures have been proposed to assess bimodality (Chasani and Likas, 2022). Here, we calculated the dip statistic, a widely used measure, to quantify the deviation of the correlation distribution from an unimodal distribution (Hartigan and Hartigan, 1985). Then the dip statistic was correlated with Psig-B using a linear regression model. The resulting t-statistics associated with the bimodality measure quantified the contribution of the bimodality to the Psig-B and the R-squared of the model quantified the variances in Psig-B explained by the bimodality. As the supplementary analysis, we repeated the analysis using the distance between the positive and negative modes as a measure of bimodality. This approach provided improved visualization of the trends between Psig-B and bimodality.

### 2.6. Supplementary analysis

A list of supplementary analyses was performed to assess the robustness of the main findings and explore novel analytical approaches.Table S2 provides details of the settings for Analyses S1 to S17.

Analyses S1 and S2 repeated the main analysis using simulated brain maps with different profiles of spatial autocorrelations with Moran’s I centered around 0.01 and 0.02. The analyses aimed to assess the robustness of the main findings across different profiles of spatial autocorrelations in the simulated brain maps.

Analysis S3 repeated the main analysis using realistic gene sets associated with molecular functions from the Gene Ontology (GO) knowledge base (Ashburner, et al., 2000). The randomly simulated gene sets used in the main analysis might not represent realistic gene sets typically selected due to being related to some molecular components based on researchers’ interests. The purpose of Analysis S3 was to assess the performance of the main analysis in a more realistic context. Specifically, the GO database was obtained using the R package org.Hs.eg.db (version 3.13). GO gene sets with sizes ranging from 20 to 200 genes were selected, resulting in 316 gene sets that were entered into the analysis.

Analysis S4 used a lower threshold of r_donors_ > 0.2 to assess the robustness of the main findings. Genes with a similarity greater than a threshold of r_donors_ > 0.2 across donors were included, resulting in a total of 12,668 genes across cortical regions (i.e., background genes) for the subsequent analysis. The preprocessing steps were the same as described above and the preprocessed data, provided by the ENIGMA Toolbox (Larivière, et al., 2021), can be accessed at (https://github.com/saratheriver/enigma-extra).

Analyses S5 and S6 evaluated the performance of the coexpression-matched (i.e., null-expressed-gene) and brain-specific (i.e., null-brain-gene) competitive null models and competitive null models (i.e., null-brain-gene). The coexpression-matched competitive null models were generated by sampling genes exhibiting similar co-expression patterns to those in the empirical model, while the brain-specific competitive null models were created by sampling genes with strong expression in brain tissue relative to other body tissues. Details for the identification of brain-specific genes can be found in the previous study (Wei, et al.,2022). These two types of null models have been proposed to improve the performance of the competitive null models in the previous study (Wei, et al., 2022). In this study, the transcriptional profiles were averaged within gene sets and correlated with brain maps, which did not involve aggregating associations within gene sets (i.e., aggregation-independent). In comparison, the analytical strategies examined in our study involved aggregating associations within gene sets (i.e., aggregation-dependent). The purpose of the analyses was to assess the applicability and efficacy of these models in the context of aggregation-dependent scenarios.

Analyses S7 and S8 repeated the main analysis with the simulated brain data within the Schaefer100 (50 left-hemisphere parcels) and Schaefer200 (100 left-hemisphere parcels) parcellations. These parcellations were functionally-defined and consisted of equally-sized parcels (Schaefer, et al., 2018). The purpose of the analyses was to assess whether the findings of the main analysis were replicated using alternative parcellations.

For the simulation of brain maps within the Schaefer100 and Schaefer200 parcellations, we first examined the performance of Moran’s I by simulating 1000 random brain maps by drawing each regional value independently from a uniform distribution ranging from −1 to 1. In this simulation, the averaged Moran’s I (i.e., a measure of spatial autocorrelation) was centered around the expected value of no spatial autocorrelation (i.e., −1/(50–1)=−0.02 for Schaefer100 and −1/(100–1)=−0.01 for Schaefer200 respectively), suggesting the Moran’s I was a good indicator of the spatial dependence for these parcellations (see Analysis S18). Then, 1000 brain maps with desired spatial autocorrelation (Moran’s *I* = 0.03) were simulated using the subsampling approach as described in the main analysis. The subsampling approach also offered better control over the desired Moran’s I in comparison to the Gaussian random fields approach for these two parcellations (see Analysis S18). The gene expression data for the Schaefer100 and Schaefer200 parcellations was preprocessed following the same steps as described in the main analysis. The preprocessed data, provided by the ENIGMA Toolbox (Larivière, et al., 2021), can be accessed at (https://github.com/saratheriver/enigma-extra).

Analysis S9 evaluated the robustness of the main analysis using realistic brain maps and gene sets. Realistic brain maps included six statistical maps of case-control comparisons of Desikan atlas cortical thickness in attention deficit hyperactivity disorder (ADHD; cases: 1814, controls: 1602), autism spectrum disorder (ASD; case: 1821, controls: 1823), bipolar disorder (BD; cases: 1555, controls: 3423), major depressive disorder (MDD; cases: 2695, controls: 3627), obsessive-compulsive disorder (OCD; cases: 2274, controls: 2013) and schizophrenia (SCZ; cases: 2716, controls: 3272) and one cortical map of standard loadings of the first principal component derived from the cortical thickness of 24,750 adult participants. More details about these realistic brain maps can be found in our previous study (Cao, et al., 2023). In this analysis, a smaller number of prior gene sets were used for visualization purposes. Specifically, the gene sets were taken from the SynGO database where gene annotations were manually curated (Koopmans, et al., 2019). Gene sets in the SynGO database with at least 20 genes available from the processed AHBA dataset (i.e., 6513 genes) were used, resulting in 49 realistic gene sets that were entered into the analysis.

Analyses S10, S11, S12 and S13 aimed to evaluate the performance of the partial least squares (PLS) regression for assessing associations between brain maps and transcriptional profiles. In the main analysis, we employed a univariate approach (i.e., Pearson’s correlation) to examine the associations between the brain map and transcriptional profiles. In contrast, PLS considered the multivariate structure within the datasets and enabled the identification of latent factors capturing the maximum correlation between the brain map and transcriptional profiles. In this analysis, we derived the regression coefficients from PLS regression, which represented the weights or importance assigned to each predictor variable (i.e., each gene’s expression data) in predicting the response variable (i.e., the brain map). These coefficients indicated the magnitude and direction of the effect that each predictor variable had on the response variable within each PLS component. The first PLS component explained the largest variance in the predictors (i.e., transcriptional profiles) while also maximizing the correlation with the response variable (i.e., a brain map) and it was often of particular interest in PLS regression and previous imaging transcriptomics analysis (Hansen, et al., 2022; Li, et al., 2021; Morgan, et al., 2019; Romero-Garcia, et al., 2020; Xue, et al., 2023; Zhu, et al., 2021). Thus, we focused on the PLS regression coefficients of the first component and evaluated the performance of different test statistics and null models using these coefficients. Specifically, Analyses S10 substituted Pearson’s correlation in the main analysis with the coefficients of the first PLS component. We found that the co-expression contributed to the inflated Psig in the competitive null models when using the PLS regression coefficients. Therefore, Analyses S11 and S12 further explored the possibility of utilizing co-expression-matched and brain-specific competitive null models to address the co-expression bias. Analysis S13 evaluated the performance of the PLS regression using realistic brain maps and gene sets. Details for the realistic brain maps and gene sets can be found in Analysis S9.

Analyses S14 and S15 investigated the feasibility of using model fit of the PLS regression to establish associations between a brain map and gene set transcriptional profiles. In this approach, the transcriptional profiles of a gene set were used to predict a brain map. To assess the model fit of the PLS regression, the root mean squared error of prediction (RMSEP) was calculated using a 10-fold cross-validation. The first PLS component explained the largest variance in the predictors (i.e., transcriptional profiles) while also maximizing the correlation with the response variable (brain maps) and it was often of particular interest in PLS regression and previous imaging transcriptomics analysis (Hansen, et al., 2022; Li, et al., 2021; Morgan, et al., 2019; Romero-Garcia, et al., 2020; Xue, et al., 2023; Zhu, et al., 2021). Therefore, we compared the RMSEP of the first PLS component against that of either competitive or self-contained null models to determine the significance of the gene set. This direct comparison of PLS model fit eliminated the need for aggregating associations (i.e., aggregation-independent), which goes beyond the scope of the present study. However, it remains valuable to readers who are interested in exploring its potential as a methodological approach. For a better comparison, the main results were presented alongside Analysis S14, and the results of Analysis S9 were presented alongside Analysis S15.

Analysis S16 repeated the main analysis using the leave-one-region-out correlation to assess the associations between brain maps and transcriptional profiles. In each iteration, one region was left out during the correlation of brain maps with transcriptional profiles. This process was repeated 34 times and an overall correlation estimate was derived by averaging across iterations. This approach was hypothesized to reduce the number of correlations that are driven by the matching of brain region and transcriptional profiles in a specific region, thereby reducing potential biases.

Analysis S17 repeated the main analysis using self-contained and competitive combined null models. This involved simultaneously spinning brain regions and shuffling gene labels. By integrating these two types of null models, we explored the feasibility of combining self-contained and competitive null models to enhance the assessment of associations between brain maps and transcriptional profiles.

Analysis S18 focused on examining the spatial autocorrelation of brain maps through three simulation approaches. The first approach generated 1000 random brain maps with regional values drawn from a uniform distribution, revealing a distribution of Global Moran’s I centered around the expected value for no spatial autocorrelation. This finding indicated spatial dependence among regions in the Desikan, Schaefer100, and Schaefer200 atlases. The second approach utilized Gaussian random fields (GRFs) to simulate brain maps (Burt, et al., 2020; Markello and Misic, 2021), with the level of spatial autocorrelation determined by the power spectral density slope (i.e., alpha). The third approach involved simulating 1000 brain maps with spatial autocorrelation using the subsampling method. Comparisons between the second and third approaches suggested that the subsampling method provided better control over the desired Moran’s I compared to the Gaussian random fields approach.

Analyses S19 and S20 examined the relationship between gene set size and Psig-G. Initially, we explored the relationship between gene set size and Psig-G using a linear model. However, as the results suggested, there was no clear trend indicating a large gene set size was associated with inflated Psig-G. Therefore, we further investigated the interactive effects of co-expression and gene set size on Psig-G. Analysis S19 performed the analysis on 500 simulated gene sets, with sizes ranging from 20 to 200 in increments of 20, whereas Analysis S20 focused on 316 realistic gene sets relevant to molecular functions, with sizes varying from 20 to 200. Since Psig-B aggregated significance across all gene sets for a brain map, we did not perform a similar analysis on Psig-B.

## 3. Results

### 3.1. Main results

**Fig. 3.**
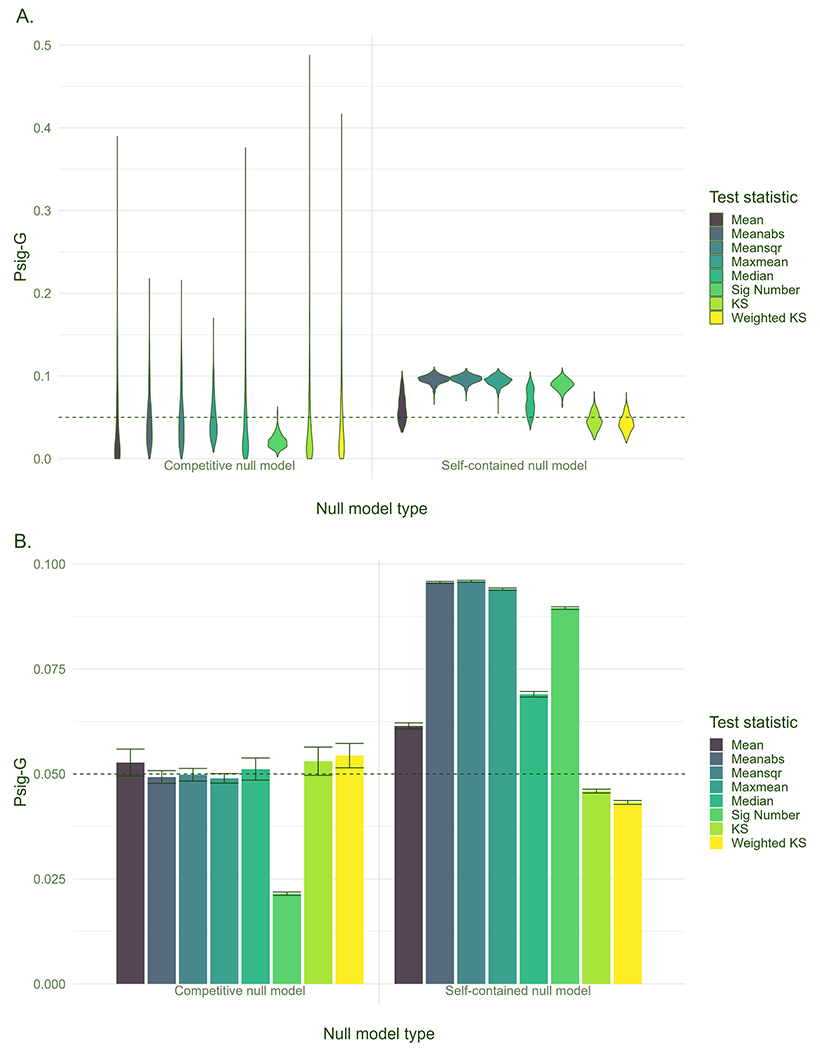
**A.** Probability of significance for each gene set (Psig-G). **B.** Mean value and standard error (i.e., standard deviation/$\sqrt{500}$) of Psig-G across all the gene sets. The tests are carried out at a nominal *α* = 0.05 significance level and the horizontal dashed line denotes a Psig-G of 0.05.

The Psig-G provided information on the likelihood of observing significant correlations between a specific gene set and a set of brain maps, with a smaller value suggesting that a lower proportion of brain maps showed a significant correlation with the gene set. As shown in Fig. 3, the mean values of Psig-G across gene sets were smaller in the competitive null models than that in the self-contained null models for most of the test statistics, except for the Psig of KS and Weighted KS. The standard error (SE) of Psig-G was greater for the competitive null models than for the self-contained null models. The Mean, Median, KS and Weighted KS showed the highest SE among all the test statistics for the competitive null models, suggesting there were false positives in which gene sets were correlated with the simulated brain maps.

**Fig. 4.**
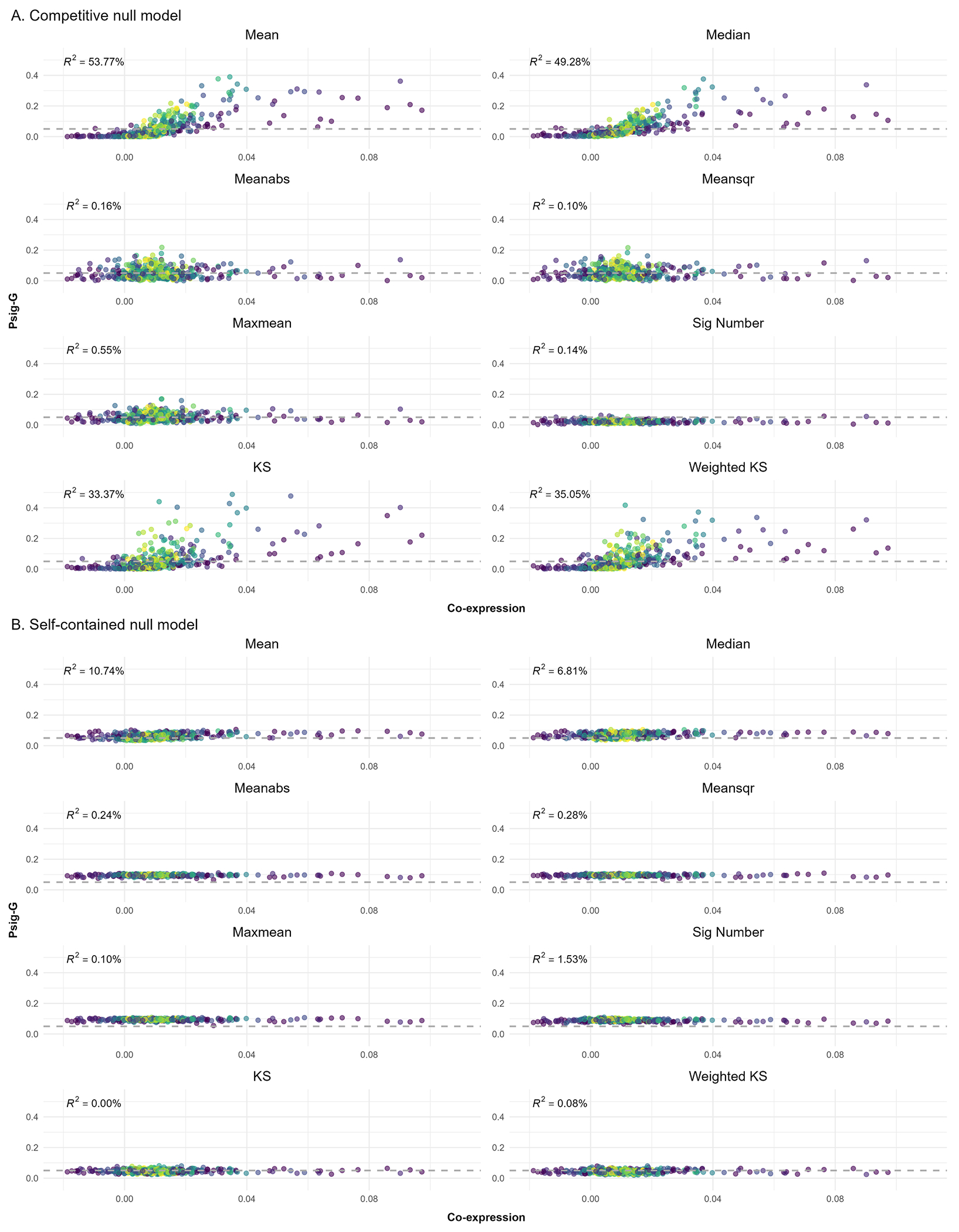
Results of co-expression analysis for the competitive (A) and self-contained null models (B). The x-axis indicates the co-expression of a specific gene set and the y-axis indicates the probability of significance for a specific gene set (Psig-G). Each dot denotes a specific gene set with the lighter color denoting the larger size of the gene set. The horizontal dashed line denotes a Psig-G of 0.05. The tests are carried out at a nominal *α* = 0.05 significance level and the horizontal dashed line denotes a Psig-G of 0.05.

As shown in Fig. 4A, Psig-G for the Mean, Median, KS and Weighted KS were positively correlated with the level of co-expression within the gene sets for the competitive null models, meaning that a gene set with high co-expression was more likely to be correlated with the simulated brain maps using these test statistics. The sign-insensitive test statistics including Meanabs, Meansqr, Maxmean and Sig Number seemed less sensitive to the co-expression in the competitive null models. By contrast, the self-contained null models yielded desirable outcomes, where the Psig-G values hovered around 0.05 with a tight SE, and there was no obvious trend between co-expression and Psig-G (see Fig. 4B). Although co-expression showed positive effects on the performance of the Mean and Median for the self-contained null models, there was no obvious point with inflated Psig-G.

**Fig. 5.**
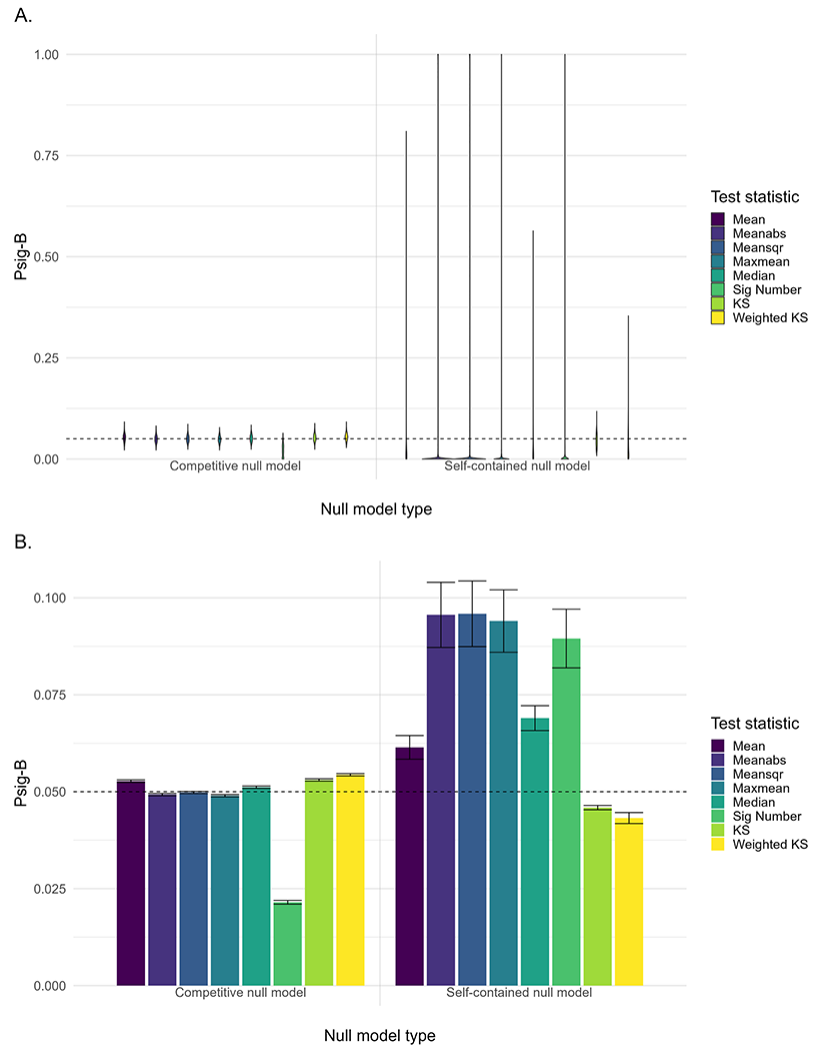
**A.** Probability of significance for each simulated brain map (Psig-B). **B.** Mean value and standard error (i.e., standard deviation/$\sqrt{1000}$) of Psig-B across all the simulated brain maps. The tests are carried out at a nominal *α* = 0.05 significance level and the horizontal dashed line denotes a Psig-B of 0.05.

The Psig-B provided information on the likelihood of observing significant correlations between a specific brain map and a set of gene sets, with a smaller value suggesting that a lower proportion of the gene sets showed a significant correlation with the brain map. As shown in Fig. 5, the mean value of Psig-B across simulated brain maps was the same as the mean value of Psig-G across gene sets, which was attributed to the fact that aggregating the results either by gene sets or brain maps did not change the final grand average. However, the SE of Psig-B across brain maps for the self-contained null models was larger than that of the competitive null models. The Meanabs, Meansqr, Maxmean and Sig Number showed the highest SE of Psig-B among all the test statistics in the self-contained null models, suggesting some brain maps were found correlated with a high proportion of the gene sets. Notably, there were 72, 74, 60 and 55 out of 1000 brain maps found to correlate with more than 80 % of the gene sets (i.e., 400 out of 500 gene sets) by these test statistics, respectively.

**Fig. 6.**
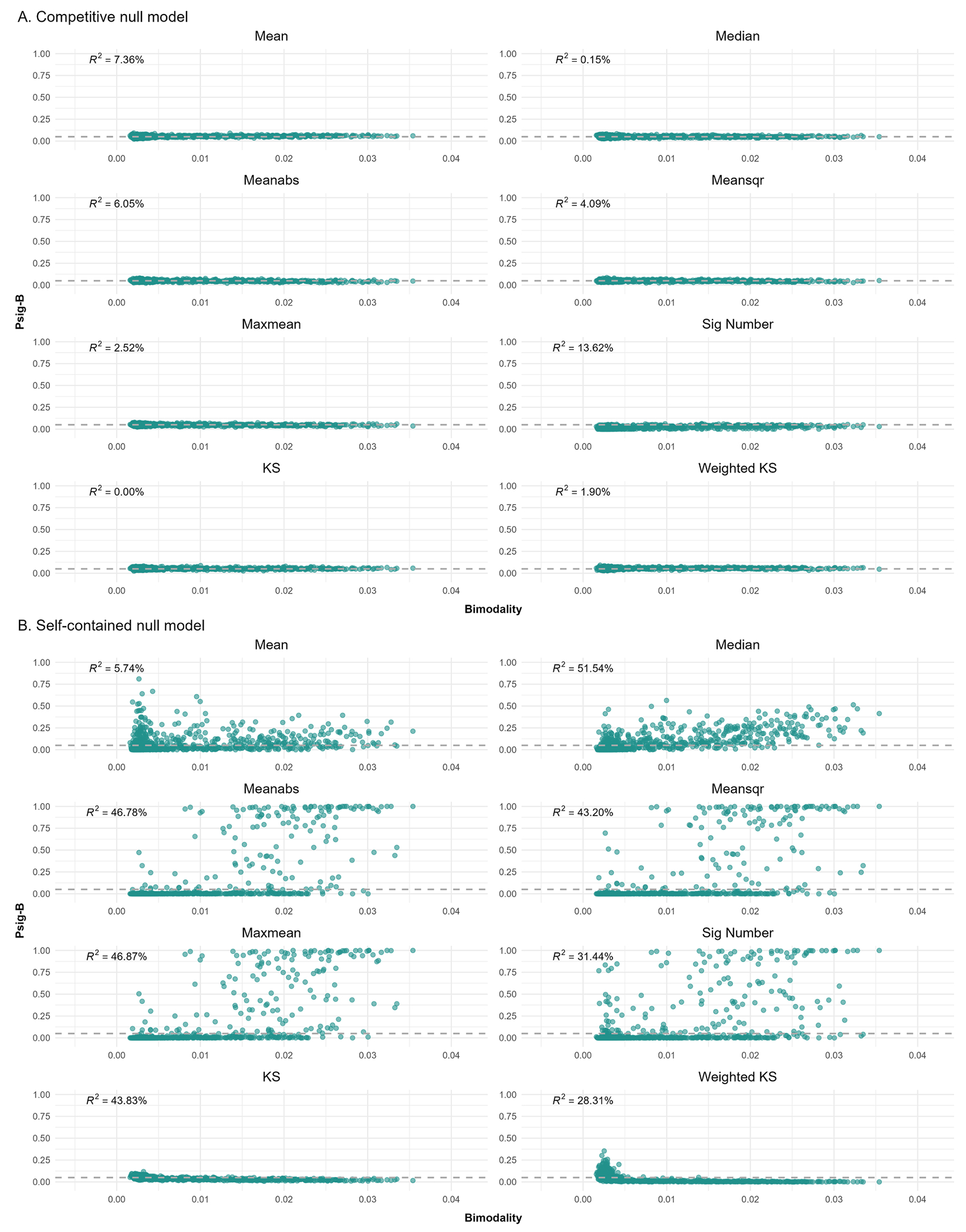
Results of the bimodality analysis for the competitive (A) and self-contained null models (B). The x-axis indicates the bimodality of the correlations between a specific brain map and transcriptional profiles of background genes, which is measured using the dip test statistic. The y-axis indicates the probability of significance for a specific brain map (Psig-B). The tests are carried out at a nominal *α* = 0.05 significance level. Each dot denotes a brain map and the horizontal dashed line denotes a Psig-B value of 0.05.

As shown in Fig. 6, the self-contained null models yielded inflated Psig-B when using Meanabs, Meansqr, Maxmean and Sig Number as the test statistic. Moreover, the bimodality of the background correlations showed a large contribution to Psig-B in these statistical tests. Although the correlation between bimodality and Psig-B was significant for the KS and Weighted KS, the effects were negative and no brain map showed an inflated Psig-B for these two test statistics. By contrast, the competitive null models yielded a desired distribution of Psig-B, where most Psig-B values hovered around 0.05 with a tight SE. The contribution of the bimodality of the background correlations to Psig-B was greatly reduced when compared to the self-contained null models.

### 3.2. Supplementary results

The above patterns (e.g., ranking of statistical methods, coexpression and bimodality bias) remained similar when using simulated brain maps with Moran’s I centered around 0.01 and 0.02 (Analyses S1 and S2), realistic gene sets of molecular functions (Analysis S3) and a lower threshold of r_donors_ > 0.2 (Analysis S4). Utilizing coexpression-matched competitive null models reduced inflated Psig-G and the associations between co-expression and Psig-G (Analysis S5). This could be partially achieved by sampling genes from brain-specific gene sets when generating competitive null models (i.e., brain-specific competitive null models; Analysis S6). However, it is worth noting that the effect of the brain-specific competitive null models seemed test statistic-dependent, which was limited for the KS and Weighted KS.

The main results were replicated when using the Schaefer100 (50 left-hemisphere parcels; Analysis S7) and Schaefer200 (100 left-hemisphere parcels; Analysis S8) parcellations. We observed that the Schaefer200 parcellation yielded a broader range of the dip-test-based measure of bimodality and a narrower range of the distance-based measure of bimodality compared to the Schaefer100 and Desikan parcellations. This suggested that the number of parcels may impact the empirical distribution of the association. However, similar trends between bimodality measures and inflated Psig-B were observed regardless of parcel numbers. For the realistic brain maps in Analysis S9, we found that the sign-insensitive test statistics yielded more significant results in self-contained null models when compared to the competitive null models, especially for effect size maps of ADHD and ASD with a high bimodality.

Analysis 10 with PLS correlations revealed similar patterns (e.g., co-expression bias) in Psig-G for the competitive null models when compared to the main analysis with Pearson’s correlations. Also, coexpression-matched and brain-specific competitive null models reduced the potentially inflated Psig-B (see Analyses S11 and S12). However, Psig-B for the self-contained null models exhibited differences when PLS correlations were used. We found that kurtosis, rather than the dip test, was sensitive to inflated Psig-B in self-contained null models. Furthermore, for the realistic data, Analysis S13 with PLS correlations reduced the number of significant terms in the self-contained null models when compared to Analysis S9 with Pearson’s correlations. Comparing the PLS model fit between empirical and null models yielded conservative results, particularly with self-contained null models (see Analyses S14 and S15). Analysis S16 with leave-one-region-out correlation revealed a similar pattern observed in the main analysis. Analysis S17, using combined self-contained and competitive null models, showed comparable outcomes to the original competitive null models for Psig-G and the original self-contained null models for Psig-B. Analysis S19 suggested that there was no clear trend between gene set size and Psig-G for both competitive and self-contained null models. However, we observed positive interactive effects between gene set size and co-expression on Psig-G for the sign-sensitive test statistics including Mean, Median, KS, and Weighted KS statistics within the competitive null models. Similar patterns were found when applying the same analysis using realistic gene sets with various sizes (Analysis S20), except that the interactive effects were found to be significant for all test statistics. It should be noted that the sign-sensitive test statistics exhibited large interactive estimates.

## 4. Discussion

The present study examined the performance of sixteen statistical tests in determining the association between gene sets and brain maps. The probability of observing significant correlations (Psig) between gene sets and brain maps was calculated for each statistical test. This was done separately for each gene set (Psig-G) and each brain map (Psig-B). The major differences between the two measures rested on what was held constant when aggregating the significant counts–gene sets for Psig-G, and brain maps for Psig-B. Although aggregating the data in different ways did not change their grand average, it revealed potential factors that could lead to inflated Psig.

### 4.1. Competitive null models

High Psig-G values were produced by the sign-sensitive test statistics including Mean, Median, KS and Weighted KS in the competitive null models, meaning that numerous gene sets were found to correlate with a high proportion of the brain maps. Further analysis revealed that the coexpression within the gene set significantly contributed to the Psig-G, suggesting that gene sets with high co-expression were more likely to be found correlated with brain maps by these test statistics. One possible explanation is that the co-expression of the resampled gene set was drawn from a larger pool of co-expressions among background genes, which could not match that of the original gene set even though the number of genes was kept the same. In the case where the original gene set had a high co-expression, its correlations with brain maps were likely clustered (e.g., positively clustered around 0.4). However, the resampled gene sets with a low co-expression would show randomly distributed correlations with brain maps (i.e., zero-centered), which would make the empirical statistic prone to exceed the null statistics. Fulcher and colleagues have shown that transcriptional associations between gene sets and brain phenotypes could be driven by the co-expression of the gene set when using the Mean as the test statistic for competitive null models (Fulcher, et al., 2021). Our results extended this by showing that several sign-sensitive test statistics, such as Median, KS and Weighted KS, were also susceptible to inflated associations driven by co-expression, while sign-insensitive test statistics including Meanabs, Meansqr, Maxmean and Sig Number produced Psig-G that did not correlate with co-expression. Of note, using Sig Number as the test statistic produced the lowest Psig-G and Psig-B for the competitive null models, suggesting that it might be conservative and could result in false negatives.

In Analyses S19 and S20, we observed no direct influence of gene set size on Psig-G. However, we did find positive interactive effects between gene set size and co-expression within the competitive null models. These findings suggest that co-expression bias within competitive null models could vary with gene set size, especially for the sign-sensitive test statistics. One possible explanation is that larger gene set sizes could lead to more credible mismatches in co-expression. These results suggested that even when the sizes of gene sets were consistent between empirical and competitive null models, nuanced interactive effects related to gene set size would persist, and these effects were particularly pronounced for sign-sensitive test statistics that were sensitive to coexpression bias.

### 4.2. Self-contained null models

Previous neuroimaging studies advocated for the use of spatial null models when examining the correspondence between two brain maps (Alexander-Bloch, et al., 2018; Markello and Misic, 2021). The efficacy of spatial null models in controlling potential false transcriptional associations driven by spatial autocorrelation has been demonstrated (Burt, et al., 2020; Fulcher, et al., 2021). Another advantage of using spatial null models was that the gene-gene relationships (i.e., their co-expression) could be preserved since the gene set was the same in each iteration of the null models. This point was demonstrated by our results that no apparent correlation between co-expression and Psig-G was observed in self-contained null models, as well as in the previous study (Fulcher, et al., 2021). The focus of the Psig-B, however, has revealed scenarios where the spinning brain approach could be suboptimal. The test statistics, such as Meansqr, Meanabs and Maxmean, were associated with inflated Psig-B with the maximum value of 1, meaning that several randomly simulated brain maps correlated with all the simulated gene sets.

By examining the bimodality of the correlations between the background genes and the brain maps, we found that brain maps with bimodal correlations with the background genes tended to be identified as correlated with more gene sets. This was likely because the correlation between a gene set and a brain map was a subsample drawn from the background correlations (i.e., correlations between the brain map and the background genes). When the background correlations were bimodal, their subsamples were more likely to be skewed. In the self-contained null models, the null background correlations were generated anew in each iteration, with only a small proportion being bimodal. As a result, their subsamples, which corresponded to null correlations between the gene sets and the spun brain maps, were more likely to be normally distributed around zero. This mismatch in the bimodality of the background correlations interacted differently with test statistics and led to varying outcomes. For instance, Median, Meanabs, Meansqr, Maxmean and Sig Number appeared to be particularly sensitive to such a scenario, resulting in inflated Psig-B values and strong correlations between the bimodality of background correlations and Psig-B values. The contribution of bimodality was lower when using Mean as the test statistic in comparison to others. Interestingly, even though bimodality negatively correlated with Psig-B of KS and Weighted KS, the performance of these two test statistics was conservative in the self-contained null models. As the KS and Weighted KS calculated cumulative sums over the ranked correlations, the ranks of the correlations were additionally examined. This was analogous to testing a competitive null hypothesis in the self-contained null models (i.e., a hybrid null model) (Goeman and Bühlmann, 2007; Maleki, et al., 2020). This hybrid approach tended to yield conservative results in the context of self-contained null models, which could potentially lead to false negatives. As the same background correlations were maintained for all iterations in the competitive null models, the contribution of the bimodality to the Psig-B was greatly reduced and there was no obvious point with inflated Psig-B in the competitive null models.

### 4.3. Addressing limitations of the null models

The competitive null models may result in false positive results driven by co-expression within gene sets, whereas self-contained null models may yield false positives if the bimodality of the background correlations is not accounted for. While both the self-contained and competitive null models have limitations, they can complement each other in mitigating the impact of these factors that affect statistical performance. The self-contained null models control for co-expression by keeping it constant across iterations, while the competitive null models control for the bimodality of the background correlations by maintaining it in each iteration. Analysis S9 with realistic brain maps and gene sets revealed that there were gene sets that survived all statistical tests, which suggests that focusing on the associations that were significant in both null models could be a feasible strategy. By retaining gene sets that were significant in both competitive and self-contained null models, this could ensure that only the most consistently significant gene categories were considered as the final results. However, this approach may lead to the omission of gene categories that were biologically meaningful but did not consistently appear in both models with different underlying hypotheses. Researchers should be aware of the trade-off between sensitivity and specificity when adopting this strategy. It is intriguing to note that building hybrid null models by simultaneously permuting brain regions and gene labels inherited the issues present in both null models (see Analysis S17), meaning that effective integration of both null models required a more sophisticated approach.

The coexpression-matched competitive null models proposed by Wei and colleagues (2012) were effective in controlling the impact of the coexpression bias in our analysis, providing a useful strategy for maintaining the same level of co-expression within the gene sets and helped reduce inflated Psig-G. The brain-specific competitive null models also showed promise by reducing Psig-G when compared to the original competitive null models. This reduction was likely attributed to the construction of null models from a smaller subset of the genes. However, it’s worth noting that the effectiveness of these brain-specific competitive null models was test statistic-dependent, particularly less effective for the KS and Weight-KS. This discrepancy suggested that while reducing the gene pool alleviated co-expression bias to some extent, it may not be as effective as the coexpression-matched approach. Moreover, our findings suggested that using sign-insensitive test statistics could be a simple yet effective approach to alleviate the impact of coexpression on the Psig-G in the competitive null models. For the self-contained null models, however, efficiently maintaining the distribution bimodality of the empirical null models requires further investigation. Conducting post hoc analysis to demonstrate the absence of a strong mismatch in bimodality could be a potential remedy and provide support for the results.

### 4.4. Partial least squares regression

In addition to univariate analysis, previous studies have employed multivariate analysis, specifically PLS, to examine the associations between brain maps and molecular profiles (Hansen, et al., 2022; Li, et al., 2021; Morgan, et al., 2019; Romero-Garcia, et al., 2020; Xue, et al., 2023; Zhu, et al., 2021). These studies mainly focused on significant genes identified by PLS, conducting over-representation analysis (ORA) (Li, et al., 2021; Morgan, et al., 2019; Romero-Garcia, et al., 2020; Xue, et al., 2023; Zhu, et al., 2021). In the supplementary analyses with PLS correlations, we took a different approach by considering all genes’ regression coefficients from the first PLS component, regardless of their significance. This allowed us to detect modest yet coordinated correlations among related genes. Notably, Psig-G for competitive null models obtained from the analysis using PLS regression coefficients mirrored the co-expression bias found in the analysis using Pearson’s correlation. This similarity could be attributed to the specific scenario we investigated, where a single brain map was predicted by 6580 predictors derived from transcriptional profiles, with a focus on the first PLS component. While PLS correlation could account for the collinearities within the predictors, the aggregation of the regression coefficients from the first PLS component was also influenced by genes with similar expression that may exhibit closely aligned regression coefficients. Moreover, we found that PLS correlations within the self-contained null models reduced the number of potentially inflated Psig-B driven by the bimodality bias in simulated data and significant terms in realistic data. However, it should be noted that kurtosis, rather than the dip test, was sensitive to inflated Psig-B in self-contained null models, suggesting PLS correlation may introduce a new confounding factor by altering distribution characteristics of the background correlations. Although this effect was not evident in the seven realistic brain maps, the simulation results suggested that the self-contained null models may fail to preserve kurtosis of the background correlations, resulting in inflated Psig-B.

Instead of using the regression coefficients of the first component, an alternative way to incorporate PLS in the imaging transcriptomics analysis was by directly comparing the model fit of the PLS regression models (Hansen, et al., 2021). Although testing this aggregation-independent approach went beyond the scope of the current paper, we included the analysis for interested readers. Analyses S14 and S15 demonstrated that this approach could be too conservative, especially for the self-contained null models. This could arise from the nature of PLS, which identified latent factors within transcriptional data of the gene sets that maximized their correlation with a brain map. Even when the brain map was generated anew by spinning brain regions, the PLS model consistently found latent factors with high correlations with the null brain map. This led to a relatively good fit of the null model, making it challenging for the empirical model fit to surpass that of the null models.

While it is often believed that multivariate analysis, such as PLS, may outperform mass univariate analysis in some cases, we did not observe significant enhancements with PLS regarding co-expression and distribution characteristics biases in the scenario where a single brain map was predicted by multiple transcriptional profiles. The additional challenges introduced, such as component selection and choices of analytical strategies (e.g., performing ORA of significant genes, focusing on PLS fit, aggregating PLS regression coefficients or loading weights), could potentially complicate the analysis and interpretation. In light of these complexities, mass univariate analyses offered a more straightforward and interpretable approach. Nonetheless, the PLS could be the optimal choice for scenarios involving multiple brain maps and transcriptional profiles (e.g., Hansen et al. 2022).

### 4.5. Implications

Previous studies have explored and discussed various null models for imaging transcriptomics analysis (Burt, et al., 2020; Fulcher, et al., 2021; Wei, et al., 2022), however, discussion on the impacts of the choices of test statistics was limited. As discussed above, our study expanded upon previous findings by 1) evaluating the performance of additional potential test statistics, and 2) highlighting potential concerns associated with the use of self-contained null models. These patterns of null model limitations and their interactions with test statistics consistently emerged in various scenarios and data configurations (e.g., Analyses S1, S2, S3, S4, S7, S8 and S16).

We noted that several resources from previous studies have been made available to perform imaging transcriptomics analysis (see Table 2). The present results may offer valuable information to assist users in understanding the settings of these toolboxes and making informed statistical decisions. Wei and colleagues (the GAMBA toolbox) adopted the approach of averaging transcriptional profiles within gene sets and subsequently correlating these averaged values with brain maps (Wei, et al., 2022), which eliminated the need to aggregate associations within gene sets (i.e., aggregation-independent). In contrast, the rest of the toolboxes’ methods as well as our analysis involved computing univariate or multivariate correlations between transcriptional profiles and a brain map, followed by aggregation using different test statistics (i.e., aggregation-dependent). This approach has been utilized in numerous studies and could offer advantages, particularly when genes within the same gene set exhibit contrasting patterns of correlations with the brain map. Due to limited choices of null models and test statistics in the aggregation-dependent toolboxes, users frequently default to the predefined settings, which could elevate the risk of false positive or negative results. For instance, findings obtained from the toolbox using competitive null models and the mean as the test statistic could be influenced by co-expression bias, warranting further revisits. The use of Weighted KS in self-contained null models could yield conservative results, potentially increasing the risk of false negatives. Coexpression-matched competitive null models, initially featured in the aggregation-independent toolbox (i.e., the GAMBA toolbox), were found to be effective in aggregation-dependent scenarios. This highlights their utility in mitigating co-expression bias for the toolboxes involving competitive null models.

### 4.6. Future directions

In the present study, we assessed linear associations between brain phenotypes and transcriptional profiles, which is a common practice in the imaging transcriptomics studies. To date, exploration of non-linear associations within imaging transcriptomics has been limited. In light of this, incorporating non-linear association measures like mutual information (Cover, 1999), could be a promising future direction. Moreover, the choice of the test statistics was mainly based on their relevance and common usage in the field, as well as our interests. However, we acknowledge that our selection was not exhaustive, and there is room for the development of novel test statistics in the future. Thus, our results could serve as a benchmark, encouraging further exploration and comparison with other test statistics. We noted that using Sig Number in competitive null models and KS-based statistics in self-contained null models produced conservative results, which could potentially lead to false negatives. However, due to the absence of a ground truth, we were unable to quantify the extent of false negatives for each statistical test. Further research is needed to investigate this matter.

## 5. Conclusion

We systematically evaluated the performance of eight different test statistics in both competitive and self-contained null models for imaging transcriptomics analysis. Our results highlighted limitations in both null models, which should be acknowledged when interpreting the findings. Specifically, we found that competitive null models may produce false positives driven by co-expression within gene sets, while self-contained null models may fail to account for bimodal correlations between all available genes and brain phenotypes, leading to false positives. These potential sources of bias interacted differently with test statistics, resulting in varying outcomes. Comprehensive supplementary analyses with various configurations, including realistic scenarios, supported the main results presented in the study. Overall, the present study offers insights into the selection of statistical tests for imaging transcriptomics studies, highlighting areas for further investigation and refinement in the evaluation of novel and commonly used tests.

## Supplementary Material

1

2

3

4

5

6

7

8

9

10

11

12

13

14

15

16

17

18

19

20

21

22

23

## Figures and Tables

**Table 1.** Test statistics evaluated in the present study.

Test statistic	Description
Mean	The mean value of the correlations within the gene set
Meanabs	The mean value of the absolute correlations within the gene set
Meansqr	The mean value of the squared correlations within the gene set
Maxmean	Either the mean value of positive correlations or the mean value of negative correlations within the gene set, depending on which is greater.
Median	The median value of the correlations within the gene set
Sig Number	The number of significant (FDR-corrected) correlations within the gene sets.
Kolmogorov-Smirnov (KS)	Compute the cumulative sum for the ranked correlations between all genes and brain profiles. For each gene, increment the cumulative sum if it belongs to the gene set and decrement it otherwise. Each gene has equal weight in either the increment or the decrement, with a value of 1. The KS is calculated as the maximum deviation from zero of the cumulative sums.
Weighted KS	Same as above except that the increment or decrement is weighted by the correlation strength of the gene.

**Table 2.** Toolboxes for Imaging Transcriptomics Analysis.

Resources (Citation)	Language	Access	Null model types	Test statistics
GAMBA (Wei, et al., 2022)	MATLAB	https://github.com/dutchconnectomelab/GAMBA-MATLAB	competitive, self-contained, coexpression-matched competitive, brain-specific competitive	N/A
ABAnnotate Toolbox	MATLAB	https://github.com/LeonDLotter/ABAnnotate	self-contained	Multiple (e.g., Mean, Meanabs, Median, etc.)
GCEA (Fulcher, et al., 2021)	MATLAB	https://github.com/benfulcher/GeneCategoryEnrichmentAnalysis	competitive, self-contained	Single (Mean)
Imaging Transcriptomics Toolbox (Giacomel, et al., 2022)	Python	https://github.com/alegiac95/Imaging-transcriptomics	self-contained	Single (Weighted KS)
Virtual histology (Shin, et al., 2018)	R	SI of the paper (https://doi.org/10.1093/cercor/bhx197)	competitive	Single (Mean)

## Data Availability

The analysis was performed using R 4.1, Moran’s I was calculated using R package *ape* (Paradis and Schliep, 2019), and the dip test was performed using R package *diptest*. Computations were performed, in part, on the Vermont Advanced Computing Core. The data and code supporting the results reported in the study are available at https://github.com/zh1peng/paper_code.
