# Supplementary material for: Unraveling the molecular relevance of brain phenotypes: A comparative analysis of null models and test statistics
Source: Neuroimage. Author manuscript; Available in PMC 2024 Jun 1. (PMC11132826; doi:10.1016/j.neuroimage.2024.120622)
Supplement: 7 [file NIHMS1995015-supplement-7.zip › S13-PLS1-Realistic.html]

S13: Analysis with realistic maps and SynGO gene sets using partial least squares regression coefficients


# S13: Analysis with realistic maps and SynGO gene sets using partial least squares regression coefficients

| Analysis | Atlas (Number of regions) | Rdonor | Brain data | Gene set | Association | Null model type | Aggregation method |
| --- | --- | --- | --- | --- | --- | --- | --- |
| S13 | Desikan (34) | 0.4 | 7 realistic maps | 49 SynGO gene sets | PLS1 Coefficients | Competitive / Self-contained / Coexp-matched Competitive / Brain-specific Competitive | Mean, Meanabs, Meansqr, Maxmean, Median, KS, Weighted KS |

*Note: The significant number of associations was not feasible as an aggregation method for this analysis.*

## 0. Setup

```
project_path='F:/Google Drive/post-doc/vitural_histology_revisit/revision_code'

sim_res_path=sprintf('%s/results',project_path)
result.path=sprintf('%s/reports',project_path)

atlas='desikan'
rdonor='r0.4'
brain_type='real_brain'
gene_set_type='SynGO'
cor_type='pls1'
null_type_level=c('random_gene',
                   'spin_brain',
                   'random_gene_coexp',
                   'random_gene_subset')
null_type_label=c('Competitive null model',
                   'Self-contained null model',
                   'Coexp-matched competitive null model',
                   'Brain-specific competitive null model')
stat_level=c('mean',
            'meanabs',
            'meansqr',
            'maxmean',
            'median',
            'ks_orig',
            'ks_weighted')
stat_label=c('Mean',
            'Meanabs',
            'Meansqr',
            'Maxmean',
            'Median',
            'KS',
            'Weighted KS')
```

## 1. Load functions

```
library(knitr)
library(kableExtra)
source(sprintf('%s/functions/analysis_functions.R',project_path))
source(sprintf('%s/functions/data_functions.R',project_path))
source(sprintf('%s/functions/cor_functions.R',project_path))
```

## 2. Load Results

```
# get the list of csv files
res.files=list(
  random_gene_coexp=sprintf( '%s/Res_%s_%s_%s_%s_random_gene_coexp_%s_sim7.csv',sim_res_path,atlas,rdonor,brain_type,gene_set_type,cor_type),
  random_gene_subset=sprintf( '%s/Res_%s_%s_%s_%s_random_gene_subset_%s_sim7.csv',sim_res_path,atlas,rdonor,brain_type,gene_set_type,cor_type),
  spin_brain=sprintf( '%s/Res_%s_%s_%s_%s_spin_brain_%s_sim7.csv',sim_res_path,atlas,rdonor,brain_type,gene_set_type,cor_type),
  random_gene=sprintf('%s/Res_%s_%s_%s_%s_random_gene_%s_sim7.csv',sim_res_path,atlas,rdonor,brain_type,gene_set_type,cor_type))
# read res.files
res.df.list=lapply(res.files, read.csv, stringsAsFactors = F)

nest_by='brain'
pvals.nested=lapply(res.df.list, get_pvals_nested, nest_by=nest_by, heat_plot=F)
```

## 3. Show SynGO gene sets

```
#get order of the gene sets
gs=load_GeneSets(atlas='desikan',rdonor = 'r0.4', gs_type='SynGO')
SynGO.df=readxl::read_excel(sprintf('%s/data/GeneSets/syngo_ontologies.xlsx',project_path))
filtered_syngo=SynGO.df %>% 
              filter(`GO term ID` %in% names(gs)) %>% 
              mutate(size=map_dbl(`GO term ID`,~length(gs[[.]]))) %>% 
              select(`GO term ID`,`GO domain`,`GO term name`,size) %>%
              arrange(desc(size))
kable(filtered_syngo,caption = 'Table 1. SynGO gene sets',booktabs = T) %>% 
  kable_styling(latex_options = c("striped", "hold_position"), font_size = 10)
```

Table 1. SynGO gene sets

| GO term ID | GO domain | GO term name | size |
| --- | --- | --- | --- |
| GO:0050808 | BP | synapse organization | 178 |
| GO:0099572 | CC | postsynaptic specialization | 177 |
| SYNGO:presynprocess | BP | process in the presynapse | 156 |
| GO:0014069 | CC | postsynaptic density | 145 |
| SYNGO:postsynprocess | BP | process in the postsynapse | 126 |
| GO:0099536 | BP | synaptic signaling | 119 |
| GO:0099537 | BP | trans-synaptic signaling | 112 |
| GO:0099504 | BP | synaptic vesicle cycle | 108 |
| GO:0007268 | BP | chemical synaptic transmission | 95 |
| GO:0042734 | CC | presynaptic membrane | 83 |
| GO:0045211 | CC | postsynaptic membrane | 76 |
| GO:0098839 | CC | postsynaptic density membrane | 70 |
| GO:0099056 | CC | integral component of presynaptic membrane | 69 |
| GO:0008021 | CC | synaptic vesicle | 65 |
| GO:0099055 | CC | integral component of postsynaptic membrane | 65 |
| GO:0099072 | BP | regulation of postsynaptic membrane neurotransmitter receptor levels | 64 |
| GO:0099061 | CC | integral component of postsynaptic density membrane | 60 |
| GO:0048786 | CC | presynaptic active zone | 59 |
| GO:0030672 | CC | synaptic vesicle membrane | 58 |
| GO:0050804 | BP | modulation of chemical synaptic transmission | 56 |
| GO:0007416 | BP | synapse assembly | 53 |
| GO:0016079 | BP | synaptic vesicle exocytosis | 51 |
| GO:0048787 | CC | presynaptic active zone membrane | 39 |
| GO:0099505 | BP | regulation of presynaptic membrane potential | 35 |
| GO:0060078 | BP | regulation of postsynaptic membrane potential | 35 |
| GO:0099059 | CC | integral component of presynaptic active zone membrane | 31 |
| GO:0099092 | CC | postsynaptic density, intracellular component | 31 |
| GO:0051963 | BP | regulation of synapse assembly | 31 |
| GO:0030285 | CC | integral component of synaptic vesicle membrane | 30 |
| SYNGO:metabolism | BP | metabolism | 30 |
| GO:0099645 | BP | neurotransmitter receptor localization to postsynaptic specialization membrane | 28 |
| GO:0099173 | BP | postsynapse organization | 27 |
| GO:0099634 | CC | postsynaptic specialization membrane | 26 |
| GO:0099560 | BP | synapse adhesion between pre- and post-synapse | 26 |
| GO:0099531 | BP | presynaptic process involved in chemical synaptic transmission | 25 |
| GO:0099171 | BP | presynaptic modulation of chemical synaptic transmission | 25 |
| GO:0099060 | CC | integral component of postsynaptic specialization membrane | 24 |
| GO:0048488 | BP | synaptic vesicle endocytosis | 24 |
| GO:0099523 | CC | presynaptic cytosol | 23 |
| GO:1904315 | BP | transmitter-gated ion channel activity involved in regulation of postsynaptic membrane potential | 23 |
| GO:2000300 | BP | regulation of synaptic vesicle exocytosis | 22 |
| GO:0099563 | BP | modification of synaptic structure | 22 |
| GO:0099509 | BP | regulation of presynaptic cytosolic calcium levels | 21 |
| GO:0099175 | BP | regulation of postsynapse organization | 21 |
| GO:0099010 | BP | modification of postsynaptic structure | 21 |
| GO:1905606 | BP | regulation of presynapse assembly | 21 |
| GO:0098918 | BP | structural constituent of synapse | 20 |
| GO:0099188 | BP | postsynaptic cytoskeleton organization | 20 |
| SYNGO:transport | BP | transport | 20 |

## 4. Make heat plots

```
brainLabel=c('ADHD','ASD','BD','MDD','OCD','SCZ','PC1')
pheat.list=lapply(pvals.nested, function(x){x %>% ungroup() %>% 
                                            mutate(brainLabel=brainLabel,
                                                   pheat=pmap(list(pvals,brainLabel,list(filtered_syngo$`GO term ID`),list(stat_level),list(stat_label)),heat_plot_pvals_df), # use list to recycle fixed argument
                                            pheat_grob=map(pheat,as.grob))})
```

Figure 1. Results of transcriptional associations with realistic brain maps identified using different null models. The brain maps include effect sizes of case-control comparisons in attention deficit hyperactivity disorder (ADHD), autism spectrum disorder (ASD), bipolar disorder (BD), major depressive Disorder (MDD), obsessive-compulsive disorder (OCD), schizophrenia (SZC) and the first principal component (PC1) derived from the cortical thickness of 24,750 adult participants. Yellow squares indicate the significant associations that were determined using one of the following test statistics: Mean, Meanabs, Meansqr, Maxmean, Median, Sig Number, KS, Weighted KS, and compared against that from the different null models.

```
for (ii in c(1:length(null_type_level))){
cat('<p style="text-align: center;">', null_type_label[ii],'</p> \n')
cat('\n')
grid.arrange(grobs=pheat.list[[null_type_level[ii]]]$pheat_grob, ncol=7)
cat('\n \n')
}
```

Competitive null model

Self-contained null model

Coexp-matched competitive null model

Brain-specific competitive null model

## 5. Distribution of background correlation and measures of bimodality

### Figure 2. Distributions of background correlations for realistic brain maps. Each panel displays a histogram of correlations between a particular brain map with the transcriptional profiles of background genes, with the dip test statistic and the distance between the positive and negative modes shown as in texts. The x-axis of each histogram represents the correlation coefficient, and the y-axis represents the count of correlations falling within each bin. The ADHD, ASD, SCZ and PC1 show the clear separation between positive and negative correlations.

```
# show bg correlat
brain_info=get_brain_info(data_path=sprintf('%s/data',project_path),
                          brain_type = 'real_brain',
                          atlas = atlas,
                          rdonor = rdonor,
                          method = cor_type)


# to make density plot
gene_data=load_GeneExp(data_path=sprintf('%s/data/GeneExp',project_path),
                        atlas = atlas,
                        rdonor = rdonor)
brain_data=load_BrainDat(data_path=sprintf('%s/data/BrainDat',project_path),
                          atlas = atlas,
                          type=brain_type,
                          col_idx = 'all') 
geneList=corr_brain_gene(gene_data=gene_data, brain_data=brain_data,method=cor_type)

brain_maps = c("ADHD", "ASD", "BD", "MDD", "OCD", "SCZ", "PC1")
plist=list()
for (i in 1:7) {
  df2plot=data.frame(cor2plot=geneList[, i])
  
  # Create histogram plot
  p=ggplot(data = df2plot, aes(x = cor2plot)) +
    geom_histogram(bins = 50, fill = "grey") +
    xlim(c(-0.0005,0.0005))+
    ylim(c(0,410))+
    annotate("text", x = -0.00025, y = 400, 
             label = paste("Dip test: ", round(brain_info[i, "modetest_stat"], 3)), 
             color = "red", hjust = 0) +
    annotate("text", x = -0.00025, y = 370, 
             label = paste("Kurtosis: ", round(brain_info[i, "kurtosis"], 3)), 
             color = "red", hjust = 0)+
    ggtitle(brain_maps[i]) +
    xlab("Background associations") +
    ylab("Count") +
    theme_minimal()+
    theme(axis.title.x = element_blank(), 
          axis.title.y = element_blank(),
          plot.title = element_text(hjust = 0.5))
  
  # Add plot to list
  plist[[i]]=p
}
grid.arrange(grobs = plist, ncol = 7,
             left =textGrob("Count",gp=gpar(fontsize=12,font=2),rot=90),
             bottom=textGrob("Background associations",gp=gpar(fontsize=12,font=2)))
```
