# Supplementary material for: Unraveling the molecular relevance of brain phenotypes: A comparative analysis of null models and test statistics
Source: Neuroimage. Author manuscript; Available in PMC 2024 Jun 1. (PMC11132826; doi:10.1016/j.neuroimage.2024.120622)
Supplement: 8 [file NIHMS1995015-supplement-8.zip › S14-PLSR_Fit.html]

S14: Analysis based on model fit of partial least square (PLS) regression


# S14: Analysis based on model fit of partial least square (PLS) regression

| Analysis | Atlas (Number of regions) | Rdonor | Brain data | Gene set | Association | Null model type | Aggregation method |
| --- | --- | --- | --- | --- | --- | --- | --- |
| S14 | Desikan (34) | 0.4 | 1000 simulated maps (Moran's I=0.03) | 500 simulated gene sets | PLSR Fit | Competitive / Self-contained | NA |

*Note: For comparisons, results of the Main analysis are also included.*

## 0. Setup

```
project_path='F:/Google Drive/post-doc/vitural_histology_revisit/revision_code'

sim_res_path=sprintf('%s/results',project_path)
result.path=sprintf('%s/reports',project_path)

atlas='desikan'
rdonor='r0.4'
brain_type='sim_spatial0.03'
gene_set_type='Sim'
cor_type='pearson'
null_type_level=c('random_gene',
                   'spin_brain')
null_type_label=c('Competitive null model',
                   'Self-contained null model')
stat_level=c('mean',
            'meanabs',
            'meansqr',
            'maxmean',
            'median',
            'sig_n',
            'ks_orig',
            'ks_weighted',
            'PLSR')
stat_label=c('Mean',
            'Meanabs',
            'Meansqr',
            'Maxmean',
            'Median',
            'Sig Number',
            'KS',
            'Weighted KS',
            'PLSR')
```

## 1. Load functions

```
library(knitr)
library(kableExtra)
source(sprintf('%s/functions/analysis_functions.R',project_path))
source(sprintf('%s/functions/data_functions.R',project_path))
source(sprintf('%s/functions/cor_functions.R',project_path))
```

## 2. Load Results

```
# get the list of csv files
res.files=list(
  spin_brain=sprintf( '%s/Res_%s_%s_%s_%s_spin_brain_%s_sim1000.csv',sim_res_path,atlas,rdonor,brain_type,gene_set_type,cor_type),
  random_gene=sprintf('%s/Res_%s_%s_%s_%s_random_gene_%s_sim1000.csv',sim_res_path,atlas,rdonor,brain_type,gene_set_type,cor_type),
  plsr_spin_brain=sprintf('%s/Res_%s_%s_%s_%s_spin_brain_PLSR_sim1000.csv',sim_res_path,atlas,rdonor,brain_type,gene_set_type),
  plsr_random_gene=sprintf('%s/Res_%s_%s_%s_%s_random_gene_PLSR_sim1000.csv',sim_res_path,atlas,rdonor,brain_type,gene_set_type))
# read res.files
res.df.list=lapply(res.files, read.csv, stringsAsFactors = F)
```

## 3. Psig-G analysis

```
# Extract pvals and group them by geneSet 
# Psig-G is calculated for each gene set
nest_by='geneSet'
pvals.nested=lapply(res.df.list, get_pvals_nested, nest_by=nest_by, heat_plot=F)
psig.list=lapply(pvals.nested, get_psig, if_fdr=F)
psig.list$random_gene=merge(psig.list$random_gene, psig.list$plsr_random_gene, by=c('geneSet'))
psig.list$spin_brain=merge(psig.list$spin_brain, psig.list$plsr_spin_brain, by=c('geneSet'))
psig.list$plsr_random_gene=NULL
psig.list$plsr_spin_brain=NULL
```

### 3.1.Plot Psig-G

#### Figure 2. A. Probability of significance for each gene set (Psig-G). B. Mean value and standard error (i.e., standard deviation/√500) of Psig-G across all the gene sets.

```
p1=plot_violin_psig_list(psig.list = psig.list,
                         ylab2show = 'Psig-G',
                         title2show = 'A.',
                         title_adj = -0.07,
                         stat_level = stat_level,
                         stat_label = stat_label,
                         null_type_level = null_type_level,
                         null_type_label = null_type_label)
p2=plot_bar_psig_list(psig.list, 
                        ylab2show='Psig-G',
                        title2show = 'B.',
                        title_adj = -0.1,
                        stat_level = stat_level,
                         stat_label = stat_label,
                         null_type_level = null_type_level,
                         null_type_label = null_type_label)
grid.arrange(p1,p2,ncol=1)
```

### 3.2.Examining the correlation between co-expression and Psig-G

```
coexp_info=get_geneSetList_info(data_path=sprintf('%s/data',project_path),
                                 gs_type=gene_set_type,
                                 atlas=atlas,
                                 rdonor=rdonor)
coexp_res.nested.list=lapply(psig.list, correlate_psig_with_info,info=coexp_info,var2test='coexp_mean')
coexp_res.report.list=lapply(coexp_res.nested.list, report_res.nested)
coexp_res.plot.list=lapply(coexp_res.nested.list, 
                           plot_res.nested, 
                           xlim2show=c(-0.02,0.11),
                           annot_position=c(-0.01,0.5))
```

### 3.2.1. Plot correlation between co-expression and Psig-G

#### Figure 3. Results of co-expression analysis for the competitive (A) and self-contained null model (B). The x-axis indicates the co-expression of a specific gene set and the y-axis indicates the probability of significance for a specific gene set (Psig-G). Each dot denotes a specific gene set with the lighter color denoting the larger size of the gene set. The horizontal dashed line denotes a Psig-G of 0.05.

```
p3=grid.arrange(grobs=coexp_res.plot.list[[null_type_level[1]]],
                ncol=2,
                top = textGrob(sprintf("A. %s",null_type_label[1]),gp=gpar(fontsize=16,font=1),x = -0.01, hjust = 0),
                left =textGrob("Psig-G",gp=gpar(fontsize=12,font=2),rot=90),
                bottom=textGrob("Co-expression",gp=gpar(fontsize=12,font=2)))
p4=grid.arrange(grobs=coexp_res.plot.list[[null_type_level[2]]],
                ncol=2,
                top = textGrob(sprintf("B. %s",null_type_label[2]),gp=gpar(fontsize=16,font=1),x = -0.01, hjust = 0),
                left =textGrob("Psig-G",gp=gpar(fontsize=12,font=2),rot=90),
                bottom=textGrob("Co-expression",gp=gpar(fontsize=12,font=2)))
grid.arrange(p3,p4)
```

### 3.2.2. Report correlation between co-expression and Psig-G

```
df1=coexp_res.report.list[[null_type_level[1]]]
df2=coexp_res.report.list[[null_type_level[2]]]
kable(df1,caption = sprintf("A. %s",null_type_label[1]))%>%
  kable_styling(full_width = FALSE, position = "float_left")
kable(df2,caption = sprintf("B. %s",null_type_label[2]))%>%
  kable_styling(full_width = FALSE, position = "left")
```

A. Competitive null model

| Test statistic | t value | p value | FDR p value | R-squared |
| --- | --- | --- | --- | --- |
| Mean | 24.0679967 | 0.0000000 | 0.0000000 | 53.77% |
| Median | 21.9955237 | 0.0000000 | 0.0000000 | 49.28% |
| Meanabs | 0.8969476 | 0.3701803 | 0.4485661 | 0.16% |
| Meansqr | 0.7099938 | 0.4780403 | 0.4780403 | 0.10% |
| Maxmean | 1.6669401 | 0.0961551 | 0.1730791 | 0.55% |
| sig\_n | 0.8446257 | 0.3987254 | 0.4485661 | 0.14% |
| KS | 15.7917695 | 0.0000000 | 0.0000000 | 33.37% |
| Weighted KS | 16.3928717 | 0.0000000 | 0.0000000 | 35.05% |
| PLSR | 1.3228738 | 0.1864845 | 0.2797267 | 0.35% |

B. Self-contained null model

| Test statistic | t value | p value | FDR p value | R-squared |
| --- | --- | --- | --- | --- |
| Mean | 7.7406988 | 0.0000000 | 0.0000000 | 10.74% |
| Median | 6.0336439 | 0.0000000 | 0.0000000 | 6.81% |
| Meanabs | -1.1010588 | 0.2714030 | 0.4071045 | 0.24% |
| Meansqr | -1.1726104 | 0.2415127 | 0.4071045 | 0.28% |
| Maxmean | -0.6980452 | 0.4854747 | 0.6069299 | 0.10% |
| sig\_n | -2.7859438 | 0.0055408 | 0.0124667 | 1.53% |
| KS | -0.1338218 | 0.8935975 | 0.8935975 | 0.00% |
| Weighted KS | 0.6140037 | 0.5394932 | 0.6069299 | 0.08% |
| PLSR | 2.8817911 | 0.0041249 | 0.0123748 | 1.64% |

## 4. Psig-B analysis

### 4.1. Plot Psig-B

#### Figure 4. A. Probability of significance for each simulated brain map (Psig-B). B. Mean value and standard error (i.e., standard deviation/√1000) of Psig-B across all the simulated brain maps.

```
p1=plot_violin_psig_list(psig.list = psig.list,
                         ylab2show = 'Psig-B',
                         title2show = 'A.',
                         title_adj = -0.086,
                         stat_level = stat_level,
                         stat_label = stat_label,
                         null_type_level = null_type_level,
                         null_type_label = null_type_label)
p2=plot_bar_psig_list(psig.list, 
                        ylab2show='Psig-B',
                        title2show = 'B.',
                        title_adj = -0.1,
                        stat_level = stat_level,
                         stat_label = stat_label,
                         null_type_level = null_type_level,
                         null_type_label = null_type_label)
grid.arrange(p1,p2,ncol=1)
```

### 4.2. Examine the correlation between BI-dip and Psig-B

```
# `var2test='pos_neg_dist'` is for BI-dist `var2test='modetest_stat'` is for BI-dip. default method is dip test
brain_info=get_brain_info(data_path=sprintf('%s/data',project_path),
                          atlas=atlas,
                          rdonor=rdonor,
                          brain_type=brain_type,
                          method=cor_type)
brain_res.nested.list=lapply(psig.list, correlate_psig_with_info,info=brain_info,var2test='modetest_stat')
brain_res.report.list=lapply(brain_res.nested.list, report_res.nested)
brain_res.plot.list=lapply(brain_res.nested.list,plot_res.nested,
                           annot_position=c(0,0.95),
                           xlim2show=c(-0.005,0.042),
                           ylim2show=c(-0.05,1))
```

### 4.2.1. Plot correlation between Psig-B and BI-dip

#### Figure 5. Results of the bimodality analysis for the competitive (A) and self-contained null model (B). The x-axis indicates the bimodality of the correlations between a specific brain map and transcriptional profiles of background genes, which was measured using the dip test statistic. The y-axis indicates the probability of significance for a specific brain map (Psig-B). Each dot denotes a brain map and the horizontal dashed line denotes a Psig-B value of 0.05.

```
p3=grid.arrange(grobs=brain_res.plot.list[[null_type_level[1]]],
                ncol=2,
                top = textGrob(sprintf("A. %s",null_type_label[1]),gp=gpar(fontsize=16,font=1),x = -0.01, hjust = 0),
                left =textGrob("Psig-B",gp=gpar(fontsize=12,font=2),rot=90),
                bottom=textGrob("Bimodality",gp=gpar(fontsize=12,font=2)))
p4=grid.arrange(grobs=brain_res.plot.list[[null_type_level[2]]],
                ncol=2,
                top = textGrob(sprintf("B. %s",null_type_label[2]),gp=gpar(fontsize=16,font=1),x = -0.01, hjust = 0),
                left =textGrob("Psig-B",gp=gpar(fontsize=12,font=2),rot=90),
                bottom=textGrob("Bimodality",gp=gpar(fontsize=12,font=2)))
grid.arrange(p3,p4)
```

### 4.2.2. Report correlation between Psig-B and BI-dip

```
df1=brain_res.report.list[[null_type_level[1]]]
df2=brain_res.report.list[[null_type_level[2]]]
kable(df1,caption = sprintf("A. %s",null_type_label[1]))%>%
  kable_styling(full_width = FALSE, position = "float_left")
kable(df2,caption = sprintf("B. %s",null_type_label[2]))%>%
  kable_styling(full_width = FALSE, position = "left")
```

A. Competitive null model

| Test statistic | t value | p value | FDR p value | R-squared |
| --- | --- | --- | --- | --- |
| Mean | 8.9071689 | 0.0000000 | 0.0000000 | 7.36% |
| Median | -1.2083914 | 0.2271830 | 0.2555809 | 0.15% |
| Meanabs | -8.0187480 | 0.0000000 | 0.0000000 | 6.05% |
| Meansqr | -6.5243433 | 0.0000000 | 0.0000000 | 4.09% |
| Maxmean | -5.0767215 | 0.0000005 | 0.0000007 | 2.52% |
| sig\_n | 12.5419682 | 0.0000000 | 0.0000000 | 13.62% |
| KS | 0.0172587 | 0.9862337 | 0.9862337 | 0.00% |
| Weighted KS | 4.3997636 | 0.0000120 | 0.0000154 | 1.90% |
| PLSR | 20.7596700 | 0.0000000 | 0.0000000 | 30.16% |

B. Self-contained null model

| Test statistic | t value | p value | FDR p value | R-squared |
| --- | --- | --- | --- | --- |
| Mean | 7.798405 | 0.0000000 | 0.0000000 | 5.74% |
| Median | 32.577307 | 0.0000000 | 0.0000000 | 51.54% |
| Meanabs | 29.620882 | 0.0000000 | 0.0000000 | 46.78% |
| Meansqr | 27.551312 | 0.0000000 | 0.0000000 | 43.20% |
| Maxmean | 29.672430 | 0.0000000 | 0.0000000 | 46.87% |
| sig\_n | 21.395357 | 0.0000000 | 0.0000000 | 31.44% |
| KS | -27.904158 | 0.0000000 | 0.0000000 | 43.83% |
| Weighted KS | -19.849809 | 0.0000000 | 0.0000000 | 28.31% |
| PLSR | -3.459229 | 0.0005646 | 0.0005646 | 1.18% |

### 4.3. Examine the correlation between BI-dist and Psig-B

```
# `var2test='pos_neg_dist'` is for BI-dist `var2test='modetest_stat'` is for BI-dip. default method is dip test
brain_res.nested.list=lapply(psig.list, correlate_psig_with_info,info=brain_info,var2test='pos_neg_dist')
brain_res.report.list=lapply(brain_res.nested.list, report_res.nested)
brain_res.plot.list=lapply(brain_res.nested.list,plot_res.nested, 
                           annot_position=c(0.115,0.95),
                           xlim2show=c(-0.02,1),
                           ylim2show=c(-0.05,1))
```

### 4.3.1. Plot correlation between Psig-B and BI-dist

#### Figure S7. Results of bimodality analysis for the competitive (A) and self-contained null model (B).The x-axis indicates the bimodality of the correlations between a specific brain map and transcriptional profiles of background genes. The distance between the positive and negative modes of the correlations was used as an indicator of the bimodality. The y-axis indicates the probability of observing significant correlations for a specific brain map (Psig-B). Each dot represents a simulated brain map and the horizontal dashed line denotes Psig=0.05.

```
p3=grid.arrange(grobs=brain_res.plot.list[[null_type_level[1]]],
                ncol=2,
                top = textGrob(sprintf("A. %s",null_type_label[1]),gp=gpar(fontsize=16,font=1),x = -0.01, hjust = 0),
                left =textGrob("Psig-B",gp=gpar(fontsize=12,font=2),rot=90),
                bottom=textGrob("Bimodality",gp=gpar(fontsize=12,font=2)))
p4=grid.arrange(grobs=brain_res.plot.list[[null_type_level[2]]],
                ncol=2,
                top = textGrob(sprintf("B. %s",null_type_label[2]),gp=gpar(fontsize=16,font=1),x = -0.01, hjust = 0),
                left =textGrob("Psig-B",gp=gpar(fontsize=12,font=2),rot=90),
                bottom=textGrob("Bimodality",gp=gpar(fontsize=12,font=2)))
grid.arrange(p3,p4)
```

### 4.3.2. Report correlation between Psig-B and BI-dist

```
df1=brain_res.report.list[[null_type_level[1]]]
df2=brain_res.report.list[[null_type_level[2]]]
kable(df1,caption = sprintf("A. %s",null_type_label[1]))
kable_paper(sprintf('Psig_B_dist_%s.csv',null_type_label[1]))
kable(df2,caption = sprintf("B. %s",null_type_label[2]))%>%
  kable_styling(full_width = FALSE, position = "left")
```

A. Competitive null model

| Test statistic | t value | p value | FDR p value | R-squared |
| --- | --- | --- | --- | --- |
| Mean | 8.7283296 | 0.0000000 | 0.0000000 | 7.09% |
| Median | -1.4387673 | 0.1505300 | 0.1693462 | 0.21% |
| Meanabs | -6.9320440 | 0.0000000 | 0.0000000 | 4.59% |
| Meansqr | -5.7132002 | 0.0000000 | 0.0000000 | 3.17% |
| Maxmean | -5.2183236 | 0.0000002 | 0.0000003 | 2.66% |
| sig\_n | 17.9506673 | 0.0000000 | 0.0000000 | 24.41% |
| KS | 0.1601034 | 0.8728320 | 0.8728320 | 0.00% |
| Weighted KS | 5.2278901 | 0.0000002 | 0.0000003 | 2.67% |
| PLSR | 19.0007277 | 0.0000000 | 0.0000000 | 26.57% |

B. Self-contained null model

| Test statistic | t value | p value | FDR p value | R-squared |
| --- | --- | --- | --- | --- |
| Mean | 11.324886 | 0e+00 | 0e+00 | 11.39% |
| Median | 42.393944 | 0e+00 | 0e+00 | 64.30% |
| Meanabs | 32.739020 | 0e+00 | 0e+00 | 51.78% |
| Meansqr | 31.427096 | 0e+00 | 0e+00 | 49.74% |
| Maxmean | 33.502697 | 0e+00 | 0e+00 | 52.93% |
| sig\_n | 27.161183 | 0e+00 | 0e+00 | 42.50% |
| KS | -35.652532 | 0e+00 | 0e+00 | 56.02% |
| Weighted KS | -26.124829 | 0e+00 | 0e+00 | 40.61% |
| PLSR | -4.965066 | 8e-07 | 8e-07 | 2.41% |
