# Supplementary material for: Unraveling the molecular relevance of brain phenotypes: A comparative analysis of null models and test statistics
Source: Neuroimage. Author manuscript; Available in PMC 2024 Jun 1. (PMC11132826; doi:10.1016/j.neuroimage.2024.120622)
Supplement: 13 [file NIHMS1995015-supplement-13.zip › S18-MoranI.html]

S18: S18: Comparisons between the subsampling approach and Gaussian random fields (GRFs) approach in simulating random desikan, schaefer100 and schaefer200 data with spatial autocorrelation as measured by Global Moran's I


# S18: a comparison between the subsampling approach and Gaussian random fields (GRFs) approach in simulating random Desikan data with spatial autocorrelation as measured by Global Moran's I

## 0. Setup

```
project_path='F:/Google Drive/post-doc/vitural_histology_revisit/revision_code'
data_path=sprintf('%s/data',project_path)
```

## 1. Load functions

```
library(knitr)
library(kableExtra)
library(ggplot2)
library(grid)
library(gridExtra)
library(dplyr)
source(file.path(data_path,'BrainDat/generate_brain.R'))
```

## 2. Global Moran's I for randomly stimulated brain maps

### Figure 1. Global Moran's Is for randomly stimulated brain maps. The histogram shows the distributions of the Global Moran’s I values for 1000 random brain maps, which were simulated by drawing each regional value independently from a uniform distribution ranging from -1 to 1. The subplots display the results of nine simulations conducted at different random states. The vertical line indicates the mean values of the distribution. The average Moran's I for the randomly generated brain maps was centered around the expected value for no spatial autocorrelation (e.g.,desikan: -1/(34-1)=-0.03). This result suggests that the Moran's I is an effective indicator of spatial dependence among the regions for desikan, schaefer100 and schaefer200 atlas.

```
plist=list()
for (atlas_i in c('desikan','schaefer100', 'schaefer200')){

    n_region <- switch(atlas_i,
      'desikan'       = 34,
      'schaefer100'   = 50,
      'schaefer200'   = 100,
      NA_integer_
    )

for (seed in c(2022,2023,2024)) {
  set.seed(seed)
  df = matrix(runif(n_region * 1000, min = -1, max = 1), nrow = n_region)
  colnames(df)=paste0('null_',c(1:1000))
  df.moran = get_column_moran_idx(atlas=atlas_i,df=df)
  hist_data = hist(df.moran$moran_idx, breaks = 50, plot = FALSE)
  mu = mean(df.moran$moran_idx) # calculate the mean
  s_d=sd(df.moran$moran_idx)
  p=ggplot(data = df.moran, aes(x = moran_idx)) +
    geom_histogram(bins = 50, fill = "grey") +
    geom_vline(xintercept = mu, color = "red", linewidth = 1) +
    annotate("text", x = mu+0.005, y = 85, 
             label = paste("Mean:", round(mu, 4)), 
             color = "red", hjust = 0) +
    annotate("text", x = mu+0.005, y = 85 * 0.90, 
             label = paste("Sd:", round(s_d, 4)), 
             color = "red", hjust = 0)+
    annotate("text", x = mu+0.005, y = 85 * 0.80, 
             label = paste("Expected:", round(-1/(n_region-1), 4)), 
             color = "blue", hjust = 0)+
    ggtitle(sprintf(" %s (seed = %s)", atlas_i, seed)) +
    xlab("Global Moran's I") +
    ylab('Count')+
    ylim(c(0,90))+
    theme_minimal()
  plist[[paste0(atlas_i,as.character(seed))]] = p
 }
}

grid.arrange(grobs=plist,ncol=3)
```

## 3. Global Moran's I for brain maps that were simulated using the Gaussian random fields (GRFs) approach.

### Figure 2. Global Moran's I for brain maps that were simulated using the Gaussian random fields (GRFs) approach. More details about this approach and implementation can be found in previous studies (Burt, et al., 2020; Markello and Misic, 2021). Briefly, for each simulated brain map, the two dense, three-dimensional GRFs were created and the level of spatial autocorrelation was determined by the slope of the field's power spectral density (i.e., alpha). Then the generated GRFs were projected onto the fsaverage5 and the parcelated (i.e., desikan, schaefer100, schaefer200) data on the left hemisphere was extracted. The subplots show the results of three simulations with different power spectral density as measured by alpha. The histogram shows the distributions of the Global Moran’s Is for 1000 simulated brain maps. The vertical line indicates the mean values of the distribution.

```
plist=list()
for (atlas_i in c('desikan','schaefer100', 'schaefer200')){

    n_region <- switch(atlas_i,
      'desikan'       = 34,
      'schaefer100'   = 50,
      'schaefer200'   = 100,
      NA_integer_
    )

for (alpha_i in c(0.1,0.2,0.3)) {
  df = read.csv(sprintf('%s/GP_simulated_brain/%s_simpath/alpha_%s/alpha%s_lh_simbrain.csv',data_path,atlas_i,alpha_i,alpha_i),
                 row.names = 1)
  
  df.moran = get_column_moran_idx(atlas=atlas_i, df=df)
  
  hist_data = hist(df.moran$moran_idx, breaks = 50, plot = FALSE)
  mu = mean(df.moran$moran_idx) # calculate the mean
  s_d=sd(df.moran$moran_idx)
  p=ggplot(data = df.moran, aes(x = moran_idx)) +
    geom_histogram(bins = 50, fill = "grey") +
    geom_vline(xintercept = mu, color = "red", linewidth = 1) +
    annotate("text", x = mu+0.005, y = 70 * 0.95, 
             label = paste("Mean:", round(mu, 4)), 
             color = "red", hjust = 0) +
    annotate("text", x = mu+0.005, y = 70 * 0.90, 
             label = paste("Sd:", round(s_d, 4)), 
             color = "red", hjust = 0)+
    ggtitle(sprintf("%s (alpha = %s)", atlas_i, alpha_i)) +
    xlab("Global Moran's I") +
    ylab('Count')+
    theme_minimal()
  plist[[paste(atlas_i,alpha_i)]] = p
  }
}
grid.arrange(grobs=plist,ncol=3)
```

## 4. Global Moran's Is for brain maps that were simulated using the subsampling approach.

### Figure 3. Global Moran's Is for brain maps that were simulated using the subsampling approach. 1000 random brain maps with spatial autocorrelation were simulated by randomly drawing a subset of 1,000 brain maps with Moran’s I centered at a desired value with a standard deviation of 0.001 from 100,000 random brain maps where each regional value was independently drawn from a uniform distribution ranging from -1 to 1. The subplots show the results of different parcellation (i.e., desiakn, schaefer100, schaefer200) and target Moran’s Is (0.01, 0.02, 0.03). The histogram shows the distributions of the Global Moran’s Is for 1000 simulated brain maps. The vertical line indicates the mean values of the distribution.

```
plist=list()

for (atlas_i in c('desikan','schaefer100', 'schaefer200')){

    n_region <- switch(atlas_i,
      'desikan'       = 34,
      'schaefer100'   = 50,
      'schaefer200'   = 100,
      NA_integer_
    )
for (moran_i in c(0.01,0.02,0.03)){

  df = read.csv(sprintf('%s/BrainDat/%s_sim_spatial%s.csv',data_path,atlas_i, moran_i),
                row.names = 1)

  df.moran = get_column_moran_idx(atlas=atlas_i,df=df)
  hist_data = hist(df.moran$moran_idx, breaks = 30, plot = FALSE)
  mu = mean(df.moran$moran_idx) # calculate the mean
  s_d=sd(df.moran$moran_idx)
  p=ggplot(data = df.moran, aes(x = moran_idx)) +
    geom_histogram(bins = 30, fill = "grey") +
    geom_vline(xintercept = mu, color = "red", linewidth = 1) +
    annotate("text", x = mu*1.01, y = 75, 
             label = paste("Mean:", round(mu, 4)), 
             color = "red", hjust = 0) +
    annotate("text", x = mu*1.01, y = 75 * 0.90, 
             label = paste("Sd:", round(s_d, 4)), 
             color = "red", hjust = 0)+
    ggtitle(sprintf("%s (Moran's I=%s)",atlas_i,moran_i)) +
    xlab("Global Moran's I") +
    ylab('Count')+
    ylim(c(0,100))+
    theme_minimal()
  plist[[paste0(atlas_i,moran_i)]] = p
  }
}
grid.arrange(grobs=plist,ncol=3)
```
