# Supplementary material for: Unraveling the molecular relevance of brain phenotypes: A comparative analysis of null models and test statistics
Source: Neuroimage. Author manuscript; Available in PMC 2024 Jun 1. (PMC11132826; doi:10.1016/j.neuroimage.2024.120622)
Supplement: 14 [file NIHMS1995015-supplement-14.zip › S19-Simulated_GeneSet_Size.html]

Analysis S19: Exploring the impacts of size of simulated gene sets on Psig-G


# Analysis S19: Exploring the impacts of size of simulated gene sets on Psig-G

## 0. Setup

```
project_path='F:/Google Drive/post-doc/vitural_histology_revisit/revision_code'

sim_res_path=sprintf('%s/results',project_path)
result.path=sprintf('%s/reports',project_path)

atlas='desikan'
rdonor='r0.4'
brain_type='sim_spatial0.03'
gene_set_type='Sim'
cor_type='pearson'
null_type_level=c('random_gene',
                   'spin_brain')
null_type_label=c('Competitive null model',
                   'Self-contained null model')
stat_level=c('mean',
            'meanabs',
            'meansqr',
            'maxmean',
            'median',
            'sig_n',
            'ks_orig',
            'ks_weighted')
stat_label=c('Mean',
            'Meanabs',
            'Meansqr',
            'Maxmean',
            'Median',
            'Sig Number',
            'KS',
            'Weighted KS')
```

## 1. Load functions

```
library(knitr)
library(kableExtra)
library(nlme)
library(sjPlot)
source(sprintf('%s/functions/analysis_functions.R',project_path))
source(sprintf('%s/functions/data_functions.R',project_path))
source(sprintf('%s/functions/cor_functions.R',project_path))
```

## 2. Load Results

```
# get the list of csv files
res.files=list(
  random_gene=sprintf('%s/Res_%s_%s_%s_%s_random_gene_%s_sim1000.csv',sim_res_path,atlas,rdonor,brain_type,gene_set_type,cor_type),
  spin_brain=sprintf( '%s/Res_%s_%s_%s_%s_spin_brain_%s_sim1000.csv',sim_res_path,atlas,rdonor,brain_type,gene_set_type,cor_type))
# read res.files
res.df.list=lapply(res.files, read.csv, stringsAsFactors = F)
```

## 3.Examining the correlation between gene set size and Psig-G

```
# Extract pvals and group them by geneSet 
# Psig-G is calculated for each gene set
nest_by='geneSet'
pvals.nested=lapply(res.df.list, get_pvals_nested, nest_by=nest_by, heat_plot=F)
psig.list=lapply(pvals.nested, get_psig, if_fdr=F)
size_info=get_geneSetList_info(data_path=sprintf('%s/data',project_path),
                                 gs_type=gene_set_type,
                                 atlas=atlas,
                                 rdonor=rdonor)
size_res.nested.list=lapply(psig.list, correlate_psig_with_info,info=size_info,var2test='size')
size_res.report.list=lapply(size_res.nested.list, report_res.nested)
size_res.plot.list=lapply(size_res.nested.list, 
                           plot_res.nested, 
                           xlim2show=c(0,250),
                           annot_position=c(40,0.5))
```

### 3.1. Plot correlation between gene set size and Psig-G

#### Figure 1. Results of gene set size analysis for the competitive null model. The x-axis indicates the size of a specific gene set and the y-axis indicates the probability of significance for a specific gene set (Psig-G). Each dot denotes a specific gene set and the horizontal dashed line denotes a Psig-G of 0.05.

```
p1=grid.arrange(grobs=size_res.plot.list[[null_type_level[1]]],
                ncol=2,
                top = textGrob(sprintf("A. %s",null_type_label[1]),gp=gpar(fontsize=16,font=1),x = -0.01, hjust = 0),
                left =textGrob("Psig-G",gp=gpar(fontsize=12,font=2),rot=90),
                bottom=textGrob("Gene set size",gp=gpar(fontsize=12,font=2)))

p2=grid.arrange(grobs=size_res.plot.list[[null_type_level[2]]],
                ncol=2,
                top = textGrob(sprintf("B. %s",null_type_label[2]),gp=gpar(fontsize=16,font=1),x = -0.01, hjust = 0),
                left =textGrob("Psig-G",gp=gpar(fontsize=12,font=2),rot=90),
                bottom=textGrob("Gene set size",gp=gpar(fontsize=12,font=2)))
grid.arrange(p1,p2)
```

### 3.2. Report correlation between gene set size and Psig-G

```
df1=size_res.report.list[[null_type_level[1]]]
df2=size_res.report.list[[null_type_level[2]]]
kable(df1,caption = sprintf("A. %s",null_type_label[1]))%>%
  kable_styling(full_width = FALSE, position = "float_left")
kable(df2,caption = sprintf("B. %s",null_type_label[2]))%>%
  kable_styling(full_width = FALSE, position = "left")
```

A. Competitive null model

| Test statistic | t value | p value | FDR p value | R-squared |
| --- | --- | --- | --- | --- |
| Mean | -2.3379373 | 0.0197855 | 0.0791420 | 1.09% |
| Median | -2.0274734 | 0.0431460 | 0.1150561 | 0.82% |
| Meanabs | 0.7631211 | 0.4457525 | 0.5577822 | 0.12% |
| Meansqr | 0.6841461 | 0.4942012 | 0.5577822 | 0.09% |
| Maxmean | 0.5865345 | 0.5577822 | 0.5577822 | 0.07% |
| sig\_n | -2.3835477 | 0.0175205 | 0.0791420 | 1.13% |
| KS | -1.2346822 | 0.2175310 | 0.3480497 | 0.31% |
| Weighted KS | -1.4668359 | 0.1430519 | 0.2861038 | 0.43% |

B. Self-contained null model

| Test statistic | t value | p value | FDR p value | R-squared |
| --- | --- | --- | --- | --- |
| Mean | -6.9146785 | 0.0000000 | 0.0000000 | 8.76% |
| Median | -2.7256583 | 0.0066433 | 0.0075924 | 1.47% |
| Meanabs | 6.7672395 | 0.0000000 | 0.0000000 | 8.42% |
| Meansqr | 4.9587937 | 0.0000010 | 0.0000016 | 4.71% |
| Maxmean | 5.1320340 | 0.0000004 | 0.0000008 | 5.02% |
| sig\_n | 10.8951157 | 0.0000000 | 0.0000000 | 19.25% |
| KS | -0.6610876 | 0.5088619 | 0.5088619 | 0.09% |
| Weighted KS | -3.7878943 | 0.0001705 | 0.0002273 | 2.80% |

## 4.Examining interactive effects between gene set size and co-expression on Psig-G

```
int_res.nested.list=lapply(psig.list, correlate_psig_with_info,info=size_info,var2test='coexp_mean',int_size=TRUE)
int_res.report.list=lapply(int_res.nested.list, int_report_res.nested)
int_res.plot.list=lapply(int_res.nested.list, int_plot_res.nested)
```

### 4.1. Plot interative effects between gene set size and co-expression on Psig-G

#### Figure 2. Marginal effects of the interaction between gene set size and co-expression on Psig-G. The x-axis indicates the co-expression, and y-axis represents predicted Psig-G values for three selected gene set sizes: 50, 100, and 200.

```
p3=grid.arrange(grobs=int_res.plot.list[[null_type_level[1]]],ncol=3,
                top = textGrob(sprintf("A. %s",null_type_label[1]),gp=gpar(fontsize=16,font=1),x = -0.01, hjust = 0),
                left =textGrob("Marginal Effects on Psig-G",gp=gpar(fontsize=12,font=2),rot=90),
                bottom=textGrob("Co-expression",gp=gpar(fontsize=12,font=2)))

p4=grid.arrange(grobs=int_res.plot.list[[null_type_level[2]]],ncol=3,
                top = textGrob(sprintf("B. %s",null_type_label[2]),gp=gpar(fontsize=16,font=1),x = -0.01, hjust = 0),
                left =textGrob("Marginal Effects on Psig-G",gp=gpar(fontsize=12,font=2),rot=90),
                bottom=textGrob("Co-expression",gp=gpar(fontsize=12,font=2)))
grid.arrange(p3,p4)
```

### 4.2. Report interactive effects between gene set size and Psig-G

```
df1=int_res.report.list[[null_type_level[1]]]
df2=int_res.report.list[[null_type_level[2]]]
kable(df1,caption = sprintf("A. %s",null_type_label[1]))%>%
  kable_styling(full_width = FALSE, position = "float_left")
kable(df2,caption = sprintf("B. %s",null_type_label[2]))%>%
  kable_styling(full_width = FALSE, position = "left")
```

A. Competitive null model

| Test statistic | Interaction | p value | FDR p value |
| --- | --- | --- | --- |
| Mean | 0.0567085 | 0.0000000 | 0.0000000 |
| Median | 0.0524517 | 0.0000000 | 0.0000000 |
| Meanabs | 0.0028932 | 0.2847909 | 0.3393564 |
| Meansqr | 0.0027879 | 0.2969369 | 0.3393564 |
| Maxmean | 0.0075355 | 0.0001633 | 0.0002613 |
| sig\_n | 0.0003528 | 0.6095039 | 0.6095039 |
| KS | 0.0392903 | 0.0000000 | 0.0000000 |
| Weighted KS | 0.0409639 | 0.0000000 | 0.0000000 |

B. Self-contained null model

| Test statistic | Interaction | p value | FDR p value |
| --- | --- | --- | --- |
| Mean | 0.0058345 | 0.0000004 | 0.0000034 |
| Median | 0.0050333 | 0.0000102 | 0.0000408 |
| Meanabs | 0.0004956 | 0.2382970 | 0.4924166 |
| Meansqr | 0.0001799 | 0.6562294 | 0.6562294 |
| Maxmean | 0.0004217 | 0.3833277 | 0.4924166 |
| sig\_n | 0.0004849 | 0.3284066 | 0.4924166 |
| KS | -0.0008209 | 0.3107760 | 0.4924166 |
| Weighted KS | -0.0006438 | 0.4308645 | 0.4924166 |
