# Supplementary material for: Unraveling the molecular relevance of brain phenotypes: A comparative analysis of null models and test statistics
Source: Neuroimage. Author manuscript; Available in PMC 2024 Jun 1. (PMC11132826; doi:10.1016/j.neuroimage.2024.120622)
Supplement: 15 [file NIHMS1995015-supplement-15.zip › S20-MF_GeneSet_Size.html]

Analysis S20: Exploring the impacts of size of molecular functions relevant gene sets on Psig-G


# Analysis S20: Exploring the impacts of size of molecular functions relevant gene sets on Psig-G

## 0. Setup

```
project_path='F:/Google Drive/post-doc/vitural_histology_revisit/revision_code'

sim_res_path=sprintf('%s/results',project_path)
result.path=sprintf('%s/reports',project_path)

atlas='desikan'
rdonor='r0.4'
brain_type='sim_spatial0.03'
gene_set_type='MF'
cor_type='pearson'
null_type_level=c('random_gene',
                   'spin_brain')
null_type_label=c('Competitive null model',
                   'Self-contained null model')
stat_level=c('mean',
            'meanabs',
            'meansqr',
            'maxmean',
            'median',
            'sig_n',
            'ks_orig',
            'ks_weighted')
stat_label=c('Mean',
            'Meanabs',
            'Meansqr',
            'Maxmean',
            'Median',
            'Sig Number',
            'KS',
            'Weighted KS')
```

## 1. Load functions

```
library(knitr)
library(kableExtra)
library(nlme)
library(sjPlot)
source(sprintf('%s/functions/analysis_functions.R',project_path))
source(sprintf('%s/functions/data_functions.R',project_path))
source(sprintf('%s/functions/cor_functions.R',project_path))
```

## 2. Load Results

```
# get the list of csv files
res.files=list(
  random_gene=sprintf('%s/Res_%s_%s_%s_%s_random_gene_%s_sim1000.csv',sim_res_path,atlas,rdonor,brain_type,gene_set_type,cor_type),
  spin_brain=sprintf( '%s/Res_%s_%s_%s_%s_spin_brain_%s_sim1000.csv',sim_res_path,atlas,rdonor,brain_type,gene_set_type,cor_type))
# read res.files
res.df.list=lapply(res.files, read.csv, stringsAsFactors = F)
```

## 3.Examining the correlation between gene set size and Psig-G

```
# Extract pvals and group them by geneSet 
# Psig-G is calculated for each gene set
nest_by='geneSet'
pvals.nested=lapply(res.df.list, get_pvals_nested, nest_by=nest_by, heat_plot=F)
psig.list=lapply(pvals.nested, get_psig, if_fdr=F)
size_info=get_geneSetList_info(data_path=sprintf('%s/data',project_path),
                                 gs_type=gene_set_type,
                                 atlas=atlas,
                                 rdonor=rdonor)
size_res.nested.list=lapply(psig.list, correlate_psig_with_info,info=size_info,var2test='size')
size_res.report.list=lapply(size_res.nested.list, report_res.nested)
size_res.plot.list=lapply(size_res.nested.list, 
                           plot_res.nested, 
                           xlim2show=c(0,250),
                           annot_position=c(40,0.5))
```

### 3.1. Plot correlation between gene set size and Psig-G

#### Figure 1. Results of gene set size analysis for the competitive null model. The x-axis indicates the size of a specific gene set and the y-axis indicates the probability of significance for a specific gene set (Psig-G). Each dot denotes a specific gene set and the horizontal dashed line denotes a Psig-G of 0.05.

```
p1=grid.arrange(grobs=size_res.plot.list[[null_type_level[1]]],
                ncol=2,
                top = textGrob(sprintf("A. %s",null_type_label[1]),gp=gpar(fontsize=16,font=1),x = -0.01, hjust = 0),
                left =textGrob("Psig-G",gp=gpar(fontsize=12,font=2),rot=90),
                bottom=textGrob("Gene set size",gp=gpar(fontsize=12,font=2)))

p2=grid.arrange(grobs=size_res.plot.list[[null_type_level[2]]],
                ncol=2,
                top = textGrob(sprintf("B. %s",null_type_label[2]),gp=gpar(fontsize=16,font=1),x = -0.01, hjust = 0),
                left =textGrob("Psig-G",gp=gpar(fontsize=12,font=2),rot=90),
                bottom=textGrob("Gene set size",gp=gpar(fontsize=12,font=2)))
grid.arrange(p1,p2)
```

### 3.2. Report correlation between gene set size and Psig-G

```
df1=size_res.report.list[[null_type_level[1]]]
df2=size_res.report.list[[null_type_level[2]]]
kable(df1,caption = sprintf("A. %s",null_type_label[1]))%>%
  kable_styling(full_width = FALSE, position = "float_left")
kable(df2,caption = sprintf("B. %s",null_type_label[2]))%>%
  kable_styling(full_width = FALSE, position = "left")
```

A. Competitive null model

| Test statistic | t value | p value | FDR p value | R-squared |
| --- | --- | --- | --- | --- |
| Mean | 4.242550 | 0.0000291 | 0.0000582 | 5.42% |
| Median | 4.065422 | 0.0000607 | 0.0000971 | 5.00% |
| Meanabs | 2.888672 | 0.0041376 | 0.0047287 | 2.59% |
| Meansqr | 3.200688 | 0.0015113 | 0.0020150 | 3.16% |
| Maxmean | 4.388169 | 0.0000156 | 0.0000417 | 5.78% |
| sig\_n | 1.289472 | 0.1981830 | 0.1981830 | 0.53% |
| KS | 4.875305 | 0.0000017 | 0.0000069 | 7.04% |
| Weighted KS | 5.032107 | 0.0000008 | 0.0000065 | 7.46% |

B. Self-contained null model

| Test statistic | t value | p value | FDR p value | R-squared |
| --- | --- | --- | --- | --- |
| Mean | -4.1937718 | 0.0000357 | 0.0001429 | 5.30% |
| Median | -2.2987709 | 0.0221744 | 0.0295659 | 1.66% |
| Meanabs | 3.6380567 | 0.0003210 | 0.0008561 | 4.04% |
| Meansqr | 3.3894769 | 0.0007896 | 0.0015792 | 3.53% |
| Maxmean | 2.4485416 | 0.0148903 | 0.0238244 | 1.87% |
| sig\_n | 6.7861292 | 0.0000000 | 0.0000000 | 12.79% |
| KS | -0.3949097 | 0.6931776 | 0.6931776 | 0.05% |
| Weighted KS | -1.8810530 | 0.0608899 | 0.0695884 | 1.11% |

## 4.Examining interactive effects between gene set size and co-expression on Psig-G

```
int_res.nested.list=lapply(psig.list, correlate_psig_with_info,info=size_info,var2test='coexp_mean',int_size=TRUE)
int_res.report.list=lapply(int_res.nested.list, int_report_res.nested)
int_res.plot.list=lapply(int_res.nested.list, int_plot_res.nested)
```

### 4.1. Plot interative effects between gene set size and co-expression on Psig-G

#### Figure 2. Marginal effects of the interaction between gene set size and co-expression on Psig-G. The x-axis indicates the co-expression, and y-axis represents predicted Psig-G values for three selected gene set sizes: 50, 100, and 200.

```
p3=grid.arrange(grobs=int_res.plot.list[[null_type_level[1]]],ncol=3,
                top = textGrob(sprintf("A. %s",null_type_label[1]),gp=gpar(fontsize=16,font=1),x = -0.01, hjust = 0),
                left =textGrob("Marginal Effects on Psig-G",gp=gpar(fontsize=12,font=2),rot=90),
                bottom=textGrob("Co-expression",gp=gpar(fontsize=12,font=2)))

p4=grid.arrange(grobs=int_res.plot.list[[null_type_level[2]]],ncol=3,
                top = textGrob(sprintf("B. %s",null_type_label[2]),gp=gpar(fontsize=16,font=1),x = -0.01, hjust = 0),
                left =textGrob("Marginal Effects on Psig-G",gp=gpar(fontsize=12,font=2),rot=90),
                bottom=textGrob("Co-expression",gp=gpar(fontsize=12,font=2)))
grid.arrange(p3,p4)
```

### 4.2. Report interactive effects between gene set size and Psig-G

```
df1=int_res.report.list[[null_type_level[1]]]
df2=int_res.report.list[[null_type_level[2]]]
kable(df1,caption = sprintf("A. %s",null_type_label[1]))%>%
  kable_styling(full_width = FALSE, position = "float_left")
kable(df2,caption = sprintf("B. %s",null_type_label[2]))%>%
  kable_styling(full_width = FALSE, position = "left")
```

A. Competitive null model

| Test statistic | Interaction | p value | FDR p value |
| --- | --- | --- | --- |
| Mean | 0.0527923 | 0e+00 | 0e+00 |
| Median | 0.0572092 | 0e+00 | 0e+00 |
| Meanabs | 0.0185083 | 0e+00 | 0e+00 |
| Meansqr | 0.0162772 | 0e+00 | 0e+00 |
| Maxmean | 0.0182616 | 0e+00 | 0e+00 |
| sig\_n | 0.0044303 | 3e-07 | 3e-07 |
| KS | 0.0659599 | 0e+00 | 0e+00 |
| Weighted KS | 0.0509268 | 0e+00 | 0e+00 |

B. Self-contained null model

| Test statistic | Interaction | p value | FDR p value |
| --- | --- | --- | --- |
| Mean | -0.0004495 | 0.6279810 | 0.6279810 |
| Median | -0.0013825 | 0.1346221 | 0.2692442 |
| Meanabs | 0.0003514 | 0.4039454 | 0.4616519 |
| Meansqr | 0.0004969 | 0.2156128 | 0.3449804 |
| Maxmean | 0.0004168 | 0.3434706 | 0.4579608 |
| sig\_n | 0.0009234 | 0.0432095 | 0.1406114 |
| KS | -0.0011683 | 0.0527293 | 0.1406114 |
| Weighted KS | -0.0013719 | 0.0298182 | 0.1406114 |
