# Supplementary material for: Unraveling the molecular relevance of brain phenotypes: A comparative analysis of null models and test statistics
Source: Neuroimage. Author manuscript; Available in PMC 2024 Jun 1. (PMC11132826; doi:10.1016/j.neuroimage.2024.120622)
Supplement: 16 [file NIHMS1995015-supplement-16.zip › S21-Main_Analysis_with_rnorm.html]

S21: Analysis with a different data-generating distribution (i.e., Gaussian distribution)


# S21: Analysis with a different data-generating distribution (i.e., Gaussian distribution)

| Analysis | Atlas (Number of regions) | Rdonor | Brain data | Gene set | Association | Null model type | Aggregation method |
| --- | --- | --- | --- | --- | --- | --- | --- |
| S21 | Desikan (34) | 0.4 | 1000 simulated maps (rnorm and Moran's I=0.03) | 500 simulated gene sets | Pearson Correlation | Competitive / Self-contained | Mean, Meanabs, Meansqr, Maxmean, Median, Sig Number, KS, Weighted KS |

## 0. Setup

```
project_path='F:/Google Drive/post-doc/vitural_histology_revisit/revision_code'

sim_res_path=sprintf('%s/results',project_path)
result.path=sprintf('%s/reports',project_path)

atlas='desikan'
rdonor='r0.4'
brain_type='sim_spatial0.03'
gene_set_type='Sim'
cor_type='pearson'
null_type_level=c('random_gene',
                   'spin_brain')
null_type_label=c('Competitive null model',
                   'Self-contained null model')
stat_level=c('mean',
            'meanabs',
            'meansqr',
            'maxmean',
            'median',
            'sig_n',
            'ks_orig',
            'ks_weighted')
stat_label=c('Mean',
            'Meanabs',
            'Meansqr',
            'Maxmean',
            'Median',
            'Sig Number',
            'KS',
            'Weighted KS')
```

## 1. Load functions

```
library(knitr)
library(kableExtra)
source(sprintf('%s/functions/analysis_functions.R',project_path))
source(sprintf('%s/functions/data_functions.R',project_path))
source(sprintf('%s/functions/cor_functions.R',project_path))
```

## 2. Load Results

```
# get the list of csv files
res.files=list(
  spin_brain=sprintf( '%s/Res_%s_%s_%s_%s_spin_brain_%s_sim1000_rnorm.csv',sim_res_path,atlas,rdonor,brain_type,gene_set_type,cor_type),
  random_gene=sprintf('%s/Res_%s_%s_%s_%s_random_gene_%s_sim1000_rnorm.csv',sim_res_path,atlas,rdonor,brain_type,gene_set_type,cor_type))
# read res.files
res.df.list=lapply(res.files, read.csv, stringsAsFactors = F)
```

## 3. Psig-G analysis

```
# Extract pvals and group them by geneSet 
# Psig-G is calculated for each gene set
nest_by='geneSet'
pvals.nested=lapply(res.df.list, get_pvals_nested, nest_by=nest_by, heat_plot=F)
psig.list=lapply(pvals.nested, get_psig, if_fdr=F)
```

### 3.1.Plot Psig-G

#### Figure 2. A. Probability of significance for each gene set (Psig-G). B. Mean value and standard error (i.e., standard deviation/√500) of Psig-G across all the gene sets.

```
p1=plot_violin_psig_list(psig.list = psig.list,
                         ylab2show = 'Psig-G',
                         title2show = 'A.',
                         title_adj = -0.07,
                         stat_level = stat_level,
                         stat_label = stat_label,
                         null_type_level = null_type_level,
                         null_type_label = null_type_label)
p2=plot_bar_psig_list(psig.list, 
                        ylab2show='Psig-G',
                        title2show = 'B.',
                        title_adj = -0.1,
                        stat_level = stat_level,
                         stat_label = stat_label,
                         null_type_level = null_type_level,
                         null_type_label = null_type_label)
grid.arrange(p1,p2,ncol=1)
```

### 3.2.Examining the correlation between co-expression and Psig-G

```
coexp_info=get_geneSetList_info(data_path=sprintf('%s/data',project_path),
                                 gs_type=gene_set_type,
                                 atlas=atlas,
                                 rdonor=rdonor)
coexp_res.nested.list=lapply(psig.list, correlate_psig_with_info,info=coexp_info,var2test='coexp_mean')
coexp_res.report.list=lapply(coexp_res.nested.list, report_res.nested)
coexp_res.plot.list=lapply(coexp_res.nested.list, 
                           plot_res.nested, 
                           xlim2show=c(-0.02,0.11),
                           annot_position=c(-0.01,0.5))
```

### 3.2.1. Plot correlation between co-expression and Psig-G

#### Figure 3. Results of co-expression analysis for the competitive (A) and self-contained null model (B). The x-axis indicates the co-expression of a specific gene set and the y-axis indicates the probability of significance for a specific gene set (Psig-G). Each dot denotes a specific gene set with the lighter color denoting the larger size of the gene set. The horizontal dashed line denotes a Psig-G of 0.05.

```
p3=grid.arrange(grobs=coexp_res.plot.list[[null_type_level[1]]],
                ncol=2,
                top = textGrob(sprintf("A. %s",null_type_label[1]),gp=gpar(fontsize=16,font=1),x = -0.01, hjust = 0),
                left =textGrob("Psig-G",gp=gpar(fontsize=12,font=2),rot=90),
                bottom=textGrob("Co-expression",gp=gpar(fontsize=12,font=2)))
p4=grid.arrange(grobs=coexp_res.plot.list[[null_type_level[2]]],
                ncol=2,
                top = textGrob(sprintf("B. %s",null_type_label[2]),gp=gpar(fontsize=16,font=1),x = -0.01, hjust = 0),
                left =textGrob("Psig-G",gp=gpar(fontsize=12,font=2),rot=90),
                bottom=textGrob("Co-expression",gp=gpar(fontsize=12,font=2)))
grid.arrange(p3,p4)
```

### 3.2.2. Report correlation between co-expression and Psig-G

```
df1=coexp_res.report.list[[null_type_level[1]]]
df2=coexp_res.report.list[[null_type_level[2]]]
kable(df1,caption = sprintf("A. %s",null_type_label[1]))%>%
  kable_styling(full_width = FALSE, position = "float_left")
kable(df2,caption = sprintf("B. %s",null_type_label[2]))%>%
  kable_styling(full_width = FALSE, position = "left")
```

A. Competitive null model

| Test statistic | t value | p value | FDR p value | R-squared |
| --- | --- | --- | --- | --- |
| Mean | 23.3465393 | 0.0000000 | 0.0000000 | 52.26% |
| Median | 21.7987953 | 0.0000000 | 0.0000000 | 48.83% |
| Meanabs | 0.4400228 | 0.6601115 | 0.6601115 | 0.04% |
| Meansqr | 0.5798668 | 0.5622667 | 0.6425905 | 0.07% |
| Maxmean | 1.6547068 | 0.0986140 | 0.1577825 | 0.55% |
| sig\_n | 0.7184678 | 0.4728058 | 0.6304078 | 0.10% |
| KS | 15.4070287 | 0.0000000 | 0.0000000 | 32.28% |
| Weighted KS | 16.2754891 | 0.0000000 | 0.0000000 | 34.72% |

B. Self-contained null model

| Test statistic | t value | p value | FDR p value | R-squared |
| --- | --- | --- | --- | --- |
| Mean | 6.6838516 | 0.0000000 | 0.0000000 | 8.23% |
| Median | 4.9949211 | 0.0000008 | 0.0000033 | 4.77% |
| Meanabs | 0.2960678 | 0.7673017 | 0.8419224 | 0.02% |
| Meansqr | 0.1997750 | 0.8417381 | 0.8419224 | 0.01% |
| Maxmean | -0.1995393 | 0.8419224 | 0.8419224 | 0.01% |
| sig\_n | -1.6153460 | 0.1068691 | 0.2849843 | 0.52% |
| KS | 0.5959738 | 0.5514635 | 0.8419224 | 0.07% |
| Weighted KS | 0.7190011 | 0.4724775 | 0.8419224 | 0.10% |

## 4. Psig-B analysis

### 4.1. Plot Psig-B

#### Figure 4. A. Probability of significance for each simulated brain map (Psig-B). B. Mean value and standard error (i.e., standard deviation/√1000) of Psig-B across all the simulated brain maps.

```
p1=plot_violin_psig_list(psig.list = psig.list,
                         ylab2show = 'Psig-B',
                         title2show = 'A.',
                         title_adj = -0.086,
                         stat_level = stat_level,
                         stat_label = stat_label,
                         null_type_level = null_type_level,
                         null_type_label = null_type_label)
p2=plot_bar_psig_list(psig.list, 
                        ylab2show='Psig-B',
                        title2show = 'B.',
                        title_adj = -0.1,
                        stat_level = stat_level,
                         stat_label = stat_label,
                         null_type_level = null_type_level,
                         null_type_label = null_type_label)
grid.arrange(p1,p2,ncol=1)
```

### 4.2. Examine the correlation between BI-dip and Psig-B

```
# `var2test='pos_neg_dist'` is for BI-dist `var2test='modetest_stat'` is for BI-dip. default method is dip test
brain_info=get_brain_info(data_path=sprintf('%s/data',project_path),
                          atlas=atlas,
                          rdonor=rdonor,
                          brain_type=brain_type,
                          method=cor_type)
brain_res.nested.list=lapply(psig.list, correlate_psig_with_info,info=brain_info,var2test='modetest_stat')
brain_res.report.list=lapply(brain_res.nested.list, report_res.nested)
brain_res.plot.list=lapply(brain_res.nested.list,plot_res.nested,
                           annot_position=c(0,0.95),
                           xlim2show=c(-0.005,0.042),
                           ylim2show=c(-0.05,1))
```

### 4.2.1. Plot correlation between Psig-B and BI-dip

#### Figure 5. Results of the bimodality analysis for the competitive (A) and self-contained null model (B). The x-axis indicates the bimodality of the correlations between a specific brain map and transcriptional profiles of background genes, which was measured using the dip test statistic. The y-axis indicates the probability of significance for a specific brain map (Psig-B). Each dot denotes a brain map and the horizontal dashed line denotes a Psig-B value of 0.05.

```
p3=grid.arrange(grobs=brain_res.plot.list[[null_type_level[1]]],
                ncol=2,
                top = textGrob(sprintf("A. %s",null_type_label[1]),gp=gpar(fontsize=16,font=1),x = -0.01, hjust = 0),
                left =textGrob("Psig-B",gp=gpar(fontsize=12,font=2),rot=90),
                bottom=textGrob("Bimodality",gp=gpar(fontsize=12,font=2)))
p4=grid.arrange(grobs=brain_res.plot.list[[null_type_level[2]]],
                ncol=2,
                top = textGrob(sprintf("B. %s",null_type_label[2]),gp=gpar(fontsize=16,font=1),x = -0.01, hjust = 0),
                left =textGrob("Psig-B",gp=gpar(fontsize=12,font=2),rot=90),
                bottom=textGrob("Bimodality",gp=gpar(fontsize=12,font=2)))
grid.arrange(p3,p4)
```

### 4.2.2. Report correlation between Psig-B and BI-dip

```
df1=brain_res.report.list[[null_type_level[1]]]
df2=brain_res.report.list[[null_type_level[2]]]
kable(df1,caption = sprintf("A. %s",null_type_label[1]))%>%
  kable_styling(full_width = FALSE, position = "float_left")
kable(df2,caption = sprintf("B. %s",null_type_label[2]))%>%
  kable_styling(full_width = FALSE, position = "left")
```

A. Competitive null model

| Test statistic | t value | p value | FDR p value | R-squared |
| --- | --- | --- | --- | --- |
| Mean | 6.6343744 | 0.0000000 | 0.0000000 | 4.22% |
| Median | -0.0887099 | 0.9293302 | 0.9293302 | 0.00% |
| Meanabs | -8.3302986 | 0.0000000 | 0.0000000 | 6.50% |
| Meansqr | -6.8365513 | 0.0000000 | 0.0000000 | 4.47% |
| Maxmean | -5.4758703 | 0.0000001 | 0.0000001 | 2.92% |
| sig\_n | 12.8011399 | 0.0000000 | 0.0000000 | 14.10% |
| KS | 1.1610971 | 0.2458801 | 0.2810058 | 0.13% |
| Weighted KS | 3.6086634 | 0.0003231 | 0.0004307 | 1.29% |

B. Self-contained null model

| Test statistic | t value | p value | FDR p value | R-squared |
| --- | --- | --- | --- | --- |
| Mean | 10.68251 | 0 | 0 | 10.26% |
| Median | 38.03722 | 0 | 0 | 59.18% |
| Meanabs | 33.06771 | 0 | 0 | 52.28% |
| Meansqr | 30.92470 | 0 | 0 | 48.93% |
| Maxmean | 33.37789 | 0 | 0 | 52.75% |
| sig\_n | 26.55521 | 0 | 0 | 41.40% |
| KS | -28.07253 | 0 | 0 | 44.12% |
| Weighted KS | -19.61119 | 0 | 0 | 27.82% |

### 4.3. Examine the correlation between BI-dist and Psig-B

```
# `var2test='pos_neg_dist'` is for BI-dist `var2test='modetest_stat'` is for BI-dip. default method is dip test
brain_res.nested.list=lapply(psig.list, correlate_psig_with_info,info=brain_info,var2test='pos_neg_dist')
brain_res.report.list=lapply(brain_res.nested.list, report_res.nested)
brain_res.plot.list=lapply(brain_res.nested.list,plot_res.nested, 
                           annot_position=c(0.115,0.95),
                           xlim2show=c(-0.02,1),
                           ylim2show=c(-0.05,1))
```

### 4.3.1. Plot correlation between Psig-B and BI-dist

#### Figure S7. Results of bimodality analysis for the competitive (A) and self-contained null model (B).The x-axis indicates the bimodality of the correlations between a specific brain map and transcriptional profiles of background genes. The distance between the positive and negative modes of the correlations was used as an indicator of the bimodality. The y-axis indicates the probability of observing significant correlations for a specific brain map (Psig-B). Each dot represents a simulated brain map and the horizontal dashed line denotes Psig=0.05.

```
p3=grid.arrange(grobs=brain_res.plot.list[[null_type_level[1]]],
                ncol=2,
                top = textGrob(sprintf("A. %s",null_type_label[1]),gp=gpar(fontsize=16,font=1),x = -0.01, hjust = 0),
                left =textGrob("Psig-B",gp=gpar(fontsize=12,font=2),rot=90),
                bottom=textGrob("Bimodality",gp=gpar(fontsize=12,font=2)))
p4=grid.arrange(grobs=brain_res.plot.list[[null_type_level[2]]],
                ncol=2,
                top = textGrob(sprintf("B. %s",null_type_label[2]),gp=gpar(fontsize=16,font=1),x = -0.01, hjust = 0),
                left =textGrob("Psig-B",gp=gpar(fontsize=12,font=2),rot=90),
                bottom=textGrob("Bimodality",gp=gpar(fontsize=12,font=2)))
grid.arrange(p3,p4)
```

### 4.3.2. Report correlation between Psig-B and BI-dist

```
df1=brain_res.report.list[[null_type_level[1]]]
df2=brain_res.report.list[[null_type_level[2]]]
kable(df1,caption = sprintf("A. %s",null_type_label[1]))
kable_paper(sprintf('Psig_B_dist_%s.csv',null_type_label[1]))
kable(df2,caption = sprintf("B. %s",null_type_label[2]))%>%
  kable_styling(full_width = FALSE, position = "left")
```

A. Competitive null model

| Test statistic | t value | p value | FDR p value | R-squared |
| --- | --- | --- | --- | --- |
| Mean | 6.8475497 | 0.0000000 | 0.0000000 | 4.49% |
| Median | -0.5538125 | 0.5798312 | 0.5798312 | 0.03% |
| Meanabs | -6.9765284 | 0.0000000 | 0.0000000 | 4.65% |
| Meansqr | -6.0547614 | 0.0000000 | 0.0000000 | 3.54% |
| Maxmean | -4.2568327 | 0.0000227 | 0.0000303 | 1.78% |
| sig\_n | 17.5804146 | 0.0000000 | 0.0000000 | 23.65% |
| KS | 2.0114311 | 0.0445484 | 0.0509124 | 0.40% |
| Weighted KS | 5.2741355 | 0.0000002 | 0.0000003 | 2.71% |

B. Self-contained null model

| Test statistic | t value | p value | FDR p value | R-squared |
| --- | --- | --- | --- | --- |
| Mean | 13.77986 | 0 | 0 | 15.99% |
| Median | 45.51438 | 0 | 0 | 67.49% |
| Meanabs | 36.05027 | 0 | 0 | 56.56% |
| Meansqr | 34.64954 | 0 | 0 | 54.61% |
| Maxmean | 36.94808 | 0 | 0 | 57.77% |
| sig\_n | 31.85222 | 0 | 0 | 50.41% |
| KS | -35.22150 | 0 | 0 | 55.42% |
| Weighted KS | -26.13663 | 0 | 0 | 40.63% |
